# Air temperature estimation and modeling using data driven techniques based on best subset regression model in Egypt

**DOI:** 10.1038/s41598-025-06277-2

**Published:** 2025-06-20

**Authors:** Ahmed Elbeltagi, Dinesh Kumar Vishwakarma, Okan Mert Katipoğlu, Kallem Sushanth, Salim Heddam, Bhaskar Pratap Singh, Abhishek Shukla, Vinay Kumar Gautam, Chaitanya Baliram Pande, Saddam Hussain, Subhankar Ghosh, Hossein Dehghanisanij, Ali Salem

**Affiliations:** 1 Agricultural Engineering Department, Faculty of Agriculture, Mansoura University, Mansoura, 35516 Egypt; 2 Department of Irrigation and Drainage Engineering, College of Technology, G. B. Pant University of Agriculture and Technology, Pantnagar, Uttarakhand 263145 India; 3 Department of Civil Engineering, Erzincan Binali Yıldırım University, Erzincan, 24002 Turkey; 4 Agricultural and Food Engineering Department, Indian Institute of Technology, Kharagpur, West Bengal 721302 India; 5 Faculty of Science, Agronomy Department, Hydraulics Division, University 20 Août 1955 Skikda, Route El Hadaik, BP 26, Skikda, Algeria; 6 ANDUAT-Krishi Vigyan Kendra, Haidergarh, Barabanki, Uttar Pradesh 225124 India; 7 School of Natural Resource Management, College of Post Graduate Studies in Agricultural Sciences, Central Agricultural University, Imphal, Umiam, 793103 Meghalaya India; 8 Department of Civil Engineering, School of Core Engineering, Faculty of Science, Technology and Architecture (FoSTA), Manipal University Jaipur, Jaipur, 303007 India; 9 New Era and Development in Civil Engineering Research Group, Scientific Research Center, Al-Ayen University, Thi-Qar, Nasiriyah, 64001 Iraq; 10 Department of Agricultural and Biological Engineering, Tropical Research and Education Center (TREC), University of Florida, Homeland, FL 33031 USA; 11 Department of Irrigation and Drainage, University of Agriculture Faisalabad, Faisalabad, 38000 Pakistan; 12 Agricultural Research, Education and Extension Organization, Agricultural Engineering Research Institute, P. O. Box 31585-845, Karaj, Alborz, Iran; 13 Civil Engineering Department, Faculty of Engineering, Minia University, Minia, 61111 Egypt; 14 Structural Diagnostics and Analysis Research Group, Faculty of Engineering and Information Technology, University of Pécs, Pécs, Hungary; 15 Department of Agricultural Engineering, Institute of Agricultural Sciences, Banaras Hindu University, Varanasi, Uttar Pradesh 221005 India

**Keywords:** Input selection, Machine learning, Regression analysis, Temperature forecasting, Climate sciences, Environmental sciences, Hydrology

## Abstract

Air temperature plays a critical role in estimating agricultural water requirements, hydrological processes, and the climate change impacts. This study aims to identify the most accurate forecasting model for daily minimum (T_min_) and maximum (T_max_) temperatures in a semi-arid environment. Five machine learning models—linear regression (LR), additive regression (AR), support vector machine (SVM), random subspace (RSS), and M5 pruned (M5P)—were compared for T_max_ and T_min_ forecasting in Gharbia Governorate, Egypt, using data from 1979 to 2014. The dataset was divided into 75% for training and 25% for testing. Model input combinations were selected based on best subset regression analysis, result shows the best combination was T_min(t−1)_, T_min(t−3)_, T_min(t−4)_, T_min(t−5)_, T_min(t−6)_, T_min(t−7)_, T_min(t−8)_ and T_max (t−1)_, T_max (t−2)_, T_max (t−3)_, T_max (t−4)_, T_max (t−5)_, T_max (t−6)_, T_max (t−8)_ for daily minimum maximum air temperature forecasting, respectively. The M5P model outperformed the other models in predicting both T_max_ and T_min_. For T_min_, the M5P model achieved the lowest root mean square error (RMSE) of 2.4881 °C, mean absolute error (MAE) of 1.9515, and relative absolute error (RAE) of 40.4887, alongside the highest Nash-Sutcliffe efficiency (NSE) of 0.8048 and Pearson correlation coefficient (PCC) of 0.8971. In T_max_ forecasting, M5P showed a lower RMSE of 2.7696 °C, MAE of 1.9867, RAE of 29.5440, and higher NSE of 0.8720 and R² of 0.8720. These results suggest that M5P is a robust and precise model for temperature forecasting, significantly outperforming LR, AR, RSS, and SVM models. The findings provide valuable insights for improving decision-making in areas such as water resource management, irrigation systems, and agricultural productivity, offering a reliable tool for enhancing operational efficiency and sustainability in semi-arid regions. The Friedman ANOVA and Dunn’s test confirm significant differences among temperature forecasting models. Additive Regression overestimates, while Linear Regression and SVM align closely with actual values. Random Subspace and M5P exhibit high variability, with SVM differing significantly. For maximum temperature, Random Subspace and M5P perform similarly, while SVM remains distinct.

**Supplementary Information:**

The online version contains supplementary material available at 10.1038/s41598-025-06277-2.

## Introduction

Accurate temperature forecasting is crucial for understanding future climate patterns^1^. Climate change and global warming pose significant global challenges, intensifying water-related disasters such as floods and droughts while affecting water quality^2^. A robust understanding of temperature variations aids decision-makers in mitigating climate change impacts and enhancing infrastructure resilience and sustainability^3^. While temperature is a critical variable connecting atmospheric and land surface processes in studies of hydrological, ecological, and climate change. Numerous studies have been conducted to model air temperature^4–7^consistently emphasizing the importance of accurate air temperature estimation in the fields of meteorology, hydrology, and agro-hydrology. Consequently, more robust and accurate models are required to effectively capture the nonlinear dynamics of air temperature variation^8^.

Goodale et al.^9^ employed geographical information such as latitude, longitude, and altitude as predictors for interpolating temperature and precipitation in Ireland. Ninyerola et al.^10^ used geographic information systems (GIS) to simulate and map air temperature. Satisfactory forecasting of nonlinear air temperature behaviour in time and space is critical. Reicosky et al.^11^ evaluated methods for estimating hourly air temperatures from daily maxima and minima, achieving reasonable accuracy on clear days but poor performance on overcast days. Their study recommended direct hourly temperature measurements for precise modeling but did not assess the impact of estimation errors. Sadler and Schroll^12^ expanded this research by developing an algorithm that did not rely on predefined temperature curves, outperforming existing methods in nearly half of the cases. However, its application required constructing a site-specific normalized temperature cumulative distribution function for a full year, limiting its practicality for broader use.

The urban heat island effect^13–15^air pollution^16–18^and human mortality^19^ are all closely associated with air temperature measured at 2 m above ground level in urban environments^20–22^. In high density populated areas, monitoring and forecasting of maximum (T_max_) and minimum (T_min_) air temperatures is essential due to their association with extreme events, such as heatwaves and tropical nights^23,24^. With a large population and complex infrastructure, even minor temperature variations within a city can significantly influence both human and natural environments^25,26^. Consequently, understanding and monitoring the spatiotemporal patterns of air temperature is essential. Remote sensing data, being geographically contiguous and regularly available across most of the Earth, offer superior spatial coverage compared to surface meteorological observations^27^. This advantage is particularly significant in regions where ground-based observations are insufficient for effective spatial interpolation^28^.

Radiometers onboard satellites can estimate land surface temperature (LST) using thermal infrared (TIR; 4.0–15.0 μm) channel data through the application of specific algorithms^29,30^. Numerous studies have investigated the potential of using remotely sensed land surface temperature (LST) to estimate surface air temperature^31–34^. Linear regression was utilized to directly estimate air temperature from satellite infrared thermal data^35–37^. The uncertainty in the estimates provided by regression techniques ranges from 1.0 °C to 2.6 °C. The normalized differential vegetation index (NDVI) estimates air temperature because land cover and soil conditions can impact heat exchange between the land surface and the near-surface atmosphere^38–40^.

Geographical interpolation that integrates in-situ observations with satellite-derived geographic variables represents a highly promising method for monitoring air temperature across extensive spatial and temporal scales^41–44^. This approach offers a cost-effective means of generating continuous and spatially resolved air temperature maps. Numerous studies have employed deterministic methods, such as inverse distance weighting (IDW), alongside geostatistical techniques like ordinary kriging (OK) and cokriging, as prevalent spatial interpolation methods^45–47^. Additionally, data-driven methods have been increasingly applied, particularly at smaller scales, for the spatial interpolation of meteorological variables due to their computational efficiency and high accuracy^48–50^.

To enhance the interpolation of air temperature, data-driven approaches often utilize regression models using spatial factors as predictors. One of the most extensively utilized data-driven methodologies is multi-linear regression (MLR)^51–56^. Machine learning algorithms have been integrated into spatial interpolation as computing power has increased^57–60^. Data driven artificial intelligence (AI), machine leaning (ML) and deep machine learning (DML) methodologies have demonstrated significant success in various scientific domains in recent years^54,58,59,61,62^. These approaches have been widely applied across disciplines including water resources engineering, agro-hydrology, and agro-meteorology^63–72^. A comprehensive review of such applications is beyond the scope of this paper; therefore, only a selection of pertinent literature will be discussed.

Mohammed et al.^73^ used four ML algorithms, viz., bagging (BG), random subspace (RSS), random tree (RT), and random forest (RF), in predicting agricultural and hydrological drought events in the eastern Mediterranean. Findings indicated BG as the best model, whereas the performance of RSS remains fitting. Machine learning models for estimation of daily evaporation and air temperature to predict evaporation and mean temperature, particularly in a semi-arid climate like New Delhi, it offers a promising approach for better understanding and managing water resources in similar regions^52,74^. Anaraki et al.^75^ highlights its novel integration of metaheuristic algorithms, decomposition, and machine learning for flood frequency analysis under climate change. The findings demonstrate the effectiveness of MARS, ANN, and LSSVM_WOA in downscaling precipitation and temperature, with LSSVM_WOA_WT excelling in discharge simulation. The conclusion emphasizes the impact of model selection on uncertainty, with HadCM3 exhibiting lower uncertainty at higher return periods. Kadkhodazadeh et al.^76^ presents a robust methodology for ETo prediction and uncertainty analysis under climate change and post-pandemic recovery scenarios. By integrating machine learning models and climate projections, the results highlight an increasing trend in temperature and evapotranspiration across all scenarios. The LSBoost model proved effective, with the Monte Carlo analysis identifying minimal uncertainty at Mianeh station.

Unfortunately, data-driven models (DDMs) have inherent limitations, including their empirical nature, reliance on a “black box” framework, and vulnerability to overfitting, which can compromise their generalization ability^77–80^. Artificial intelligence models that can extract data noise and outliers and reveal the non-linear relationship between geographical factors and air temperature^81^. Cho et al.^82^ compared standard interpolation algorithms to ANN for spatial interpolation of air temperature. In other studies, ANN has since been used to spatially interpolate short-term (daily and hourly) air temperature^83^. Support vetror regression (SVR) has also been used to compare the spatial interpolation of air temperature with ANN^84^. Researchers discovered that SVR outperforms ANN for the spatial interpolation of short-term air temperature^85^. Alomar et al.^86^ has applied data-driven models for atmospheric air temperature forecasting at a continental climate region. Result reveals that SVR outperforms other models for daily forecasting, while RF and Gradient Boosting Regression (GBR) excel in weekly forecasts under climate variability. Salcedo-Sanz et al.^87^ concludes that SVR outperforms other algorithms in predicting monthly mean air temperature, though its accuracy has declined in recent decades, reflecting potential changes in the relationships among predictive variables.

While machine learning (ML) has been widely used for air temperature forecasting, few studies compare multiple ML models within the same framework. Temperature significantly impacts precipitation and drought, highlighting the need for accurate forecasting models. Previous methods often suffer from low accuracy, data sensitivity, and limited adaptability to climate change. This study addresses these gaps by analyzing daily air temperature trends in Gharbia Governorate, Egypt, using multiple ML models. We focus on selecting the optimal time lag for improved forecasting. The study aims to: (1) develop novel ML-based models for daily air T_min_ and T_max_ forecasting, (2) evaluate model performance using statistical metrics, and (3) recommend the most accurate model for future water resource management.

## Materials and methods

### Study area

**Fig. 1:**
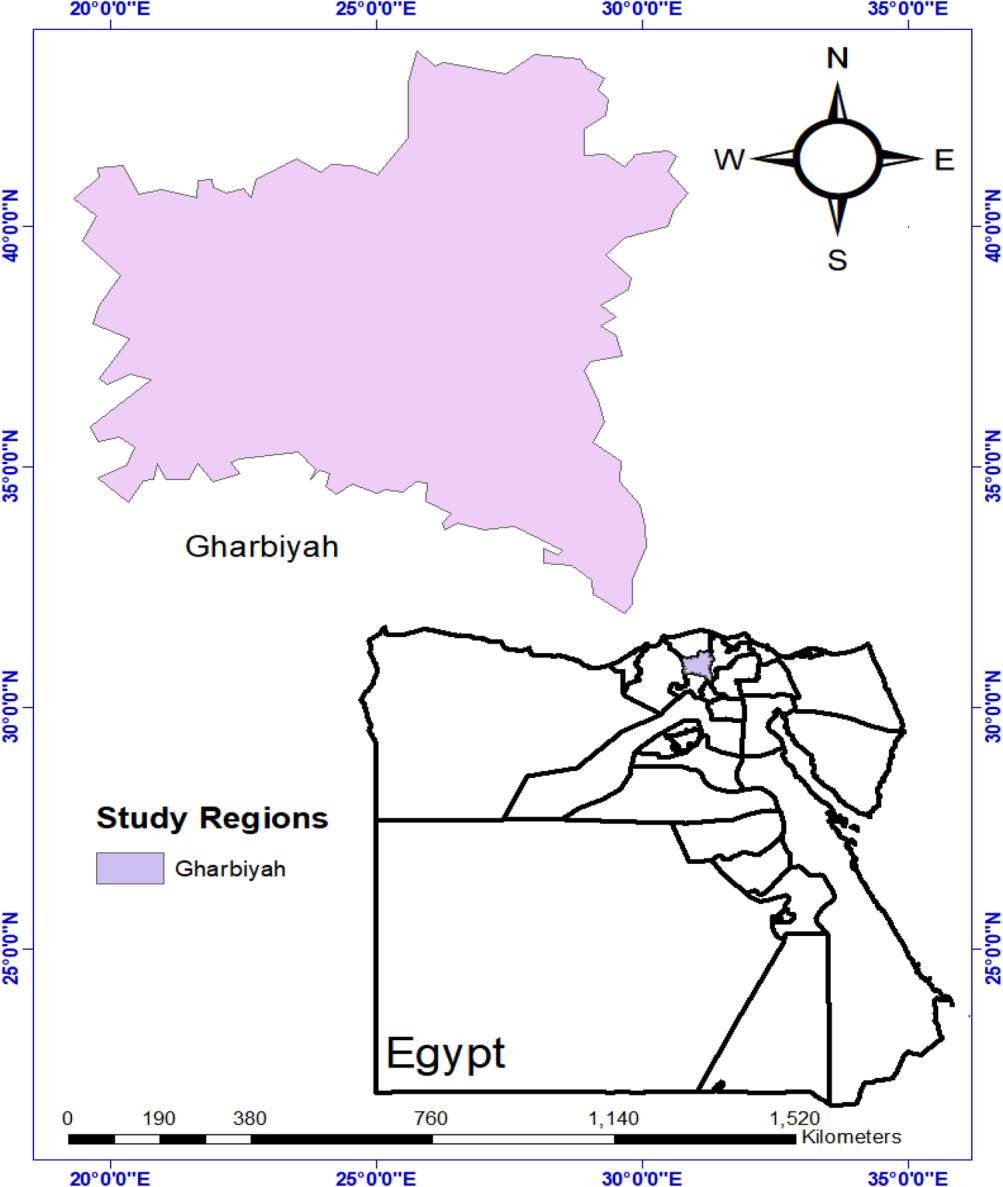
Map of study area.

The Nile Delta is Egypt’s economic and agricultural hub, comprising 11 governorates in an arid desert climate. Gharbia Governorate, located in the central Nile Delta, Egypt (30.87°N, 31.03°E) (Fig. 1), is bordered by Kafr El-Sheikh to the north, Monufia to the south, and the Damietta and Rosetta branches of the Nile to the east and west, respectively. The Nile Delta is among the world’s oldest agricultural regions. Gharbia Governorate, the study area, spans 1,942 km² and is a key agricultural and industrial center, producing cotton, rice, wheat, maize, and medicinal plants for export. Summer temperatures peak at 49.294 °C in July 2010, while winter ranges from 9 °C to 19 °C, with annual rainfall of 100–200 mm, mostly in winter. Agriculture consumes 85% of freshwater, posing a risk of future water scarcity.

### Datasets

The National Centers for Environmental Prediction (NCEP) Climate Forecast System Reanalysis (CFSR) was used to gather daily climate data variables for the examined region, such as minimum and maximum temperatures, from 1979 to 2014. The CFSR was created as a global, high-resolution, coupled system of the atmosphere, ocean, land surface, and sea ice to give the most accurate assessment of the state of these coupled domains during this time. The SWAT file format and CSV versions of the daily minimum and maximum temperatures CFSR data for the entire time were downloaded per continent. This 36-year dataset consists of 13,148 data points with no missing values during the machine learning modeling process. The dataset was pre-processed to remove outliers, reducing the total number of data points to 12,987. It was then divided into training (10,221 data points, 78.68%) and testing (2,768 data points, 21.32%) sets. Additionally, the entire training dataset was shuffled (10,221 data point) and subjected to validation to ensure model robustness. The basic statistics of training, testing, and validation datasets at study stations are presented in Table 1.

### Hyper parameter tuning for machine learning models

**Fig. 2:**
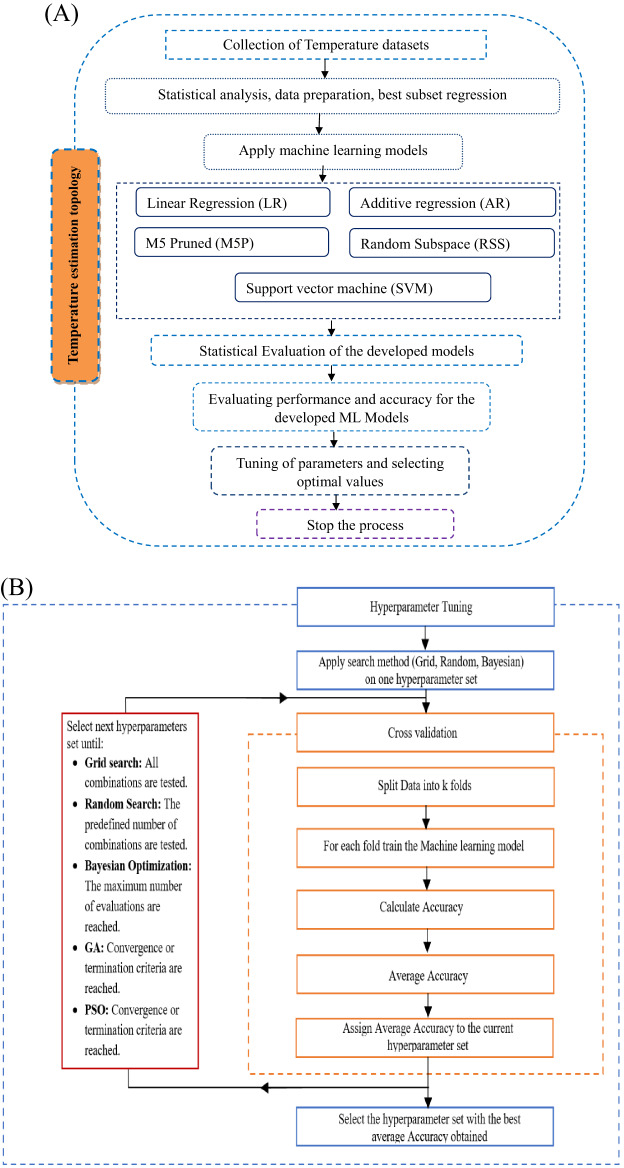
Flowchart of Temperature estimation methodology in the study area **(A)** and Hyperparameter tuning process **(B)**, (Elgeldawi et al.^86^).

Flowchart of Temperature estimation methodology in the study area is also shown in Fig. 2. The selection of hyperparameters values can affect significantly the forecasting performance independently of the machine learning technique considered. The adjustment of these parameters can be performed manually or automatically using the internal optimization option that tries to find the combination of hyperparameters for which the mean squared error per each forecasting technique is minimized (Fig. 2). The developed AI models were implemented by WEKA (Waikato Environment for Knowledge Analysis), which is a popular open-source software suite for machine learning and data mining developed at the University of Waikato in New Zealand. It is widely used for research and education, as well as in industry applications. WEKA provides a collection of machine learning algorithms for data mining tasks, along with tools for data preprocessing, classification, regression, clustering, association rule mining, and visualization.

### Best subset regression analysis

Best subset regression analysis is a statistical model selection technique that systematically evaluates all possible combinations of predictor variables to identify the optimal model based on predefined statistical criteria^52,88^. This approach is particularly useful in exploratory regression analysis, as it enables a comprehensive comparison of potential models constructed from a given set of predictors. Unlike stepwise regression methods, which iteratively add or remove predictors based on significance thresholds, best subset regression exhaustively examines every possible subset of predictors. This allows for the selection of a model that balances explanatory power with parsimony, ensuring that the chosen model maintains a high level of predictive accuracy while minimizing complexity^52,89,90^. By excluding irrelevant or redundant variables, best subset regression reduces overfitting and enhances model interpretability.

**Table 1:** The basic statistics of training, testing, and cross validation datasets at study stations.

Statistical Parameter	Minimum temperature data set			Maximum temperature data set		
	Training	Testing	Cross validation	Training	Testing	Cross validation
Mean (°C)	12.006	12.515	12.325	29.125	30.128	29.125
Standard Error (°C)	0.056	0.103	0.089	0.077	0.141	0.077
Median (°C)	12.616	13.063	14.493	30.033	30.988	30.033
Mode (°C)	16.364	11.965	15.525	35.919	14.989	35.919
Standard Deviation (°C)	5.632	5.402	4.707	7.742	7.428	7.742
Sample Variance	31.724	29.184	22.156	59.939	55.182	59.939
Kurtosis	−0.899	−0.942	−1.449	−1.020	−0.947	−1.020
Skewness	−0.286	−0.247	−0.231	−0.227	−0.235	−0.227
Range (°C)	29.577	26.848	13.500	41.356	38.852	41.356
Minimum (°C)	−5.517	−2.758	4.675	7.340	10.442	7.340
Maximum (°C)	24.060	24.090	18.175	48.696	49.294	48.696
Count	10,221	2768	10,221	10,221	2768	10,221

The primary advantage of best subset regression lies in its ability to identify models that provide robust estimates of regression coefficients while reducing prediction variance^89^. This is particularly beneficial when dealing with high-dimensional datasets, where incorporating all available predictors can lead to overfitting and decreased generalizability. The selection criteria typically used in best subset regression include metrics such as the adjusted R-squared, Akaike Information Criterion (AIC), Bayesian Information Criterion (BIC), and Mallows’ Cp, each of which provides a different perspective on model performance^51,90,91^.

Stepwise regression iteratively adds/removes predictors based on significance, balancing model simplicity and fit but risks overfitting and bias^57,71,92,93^. LASSO imposes L1 regularization, shrinking coefficients to zero for feature selection, enhancing generalizability but may bias estimates. Stepwise is computationally simpler; LASSO handles high-dimensional data better, reducing multicollinearity impact.

### Machine learning models

#### Linear regression (LR)

Linear regression analysis examines the correlation between multiple independent or predictor variables and a dependent variable. The model assumes a linear relationship between the dependent variable *Y*_*i*_ and the vector of regressors *X*_*i*_. The following Eq. 1 shows the LR Eq. 5^6^.

1 $$\:Y=\alpha\:+{\beta\:}_{1}{X}_{1}++{\beta\:}_{1}{X}_{1}$$

where a indicates the intercept, $\:{\beta\:}_{\:}$ indicates the slope, and k indicates the number of observations.

#### Additive regression (AR)

The concept of additive regression (AR) is a statistical modeling approach used to establish relationships between variables. This method offers a distinct and effective alternative by mitigating the challenges associated with high-dimensional data, commonly referred to as the curse of dimensionality. The additive model is mathematically represented as Eq. 2:

2 $$\:E\left[{y}_{i}\mid\:{x}_{i1},\dots\:,{x}_{ip}\right]={\beta\:}_{o}+\sum\limits_{j=1}^{p}\:{f}_{i}\left({x}_{ij}\right)$$

where $\:\sum\:_{j=1}^{p}\:{f}_{i}\left({x}_{ij}\right)$ shows the smooth functions fitted from the data and $\:{\beta\:}_{\varvec{o}}$ shows the regression coefficient.

#### M5 pruned (M5P)

The M5P algorithm is a regression tree-based approach used to make forecastings^94^. This regression tree consists of the root node of the entire dataset, internal nodes that create conditions based on input variables, and leaf nodes that contain linear regression. The M5P algorithm first divides the input datasets into subsets according to the datasets binary division rule^58,89,95^. Each subset is then divided into subsets based on the Least Square Deviation (LSD) function as shown in Eq. 3.

3 $$\:R\left(t\right)=\frac{1}{N\left(t\right)}\sum\limits_{i\in\:t}\:\left({y}_{i}-{y}_{m}\left(t\right)\right)$$

The LSD function calculates the internal variance at a given node $\:R\left(t\right)$, the number of units in the subset (*N*), the target variable values for each unit $\:{y}_{i}$, and the mean of the target $\:{y}_{m}$^96^.

#### Support vector machine (SVM)

SVM is a supervised learning method and was first proposed by Vapnik^97^. This method is based on statistical learning theory and is kernel-based^98^. Additionally, SVR is the SVM model used to solve various regression problems. The main purpose of this model is to reduce the error by individualizing the hyperplane. When the linear function is taken into account in this model, which is based on the tolerance limit of this model, the constraints can be reduced. As long as errors are smaller than ε, they are insignificant, and deviations are penalized (Müller et al. 2018). The SVM forecasting function (F) is given in Eq. 4.

4 $$\:F\left(x\right)=W\cdot{T}_{f}\left(x\right)+b$$

where, *W* shows the weight-age vector; $\:{T}_{f}$ indicates the nonlinear transfer function, and *b* shows the constant variable.

#### Random subspace (RSS)

RSS is an ensemble-based classification approach that constructs a forest of classifiers^99^. It operates by randomly selecting subsets of features for individual classifiers and subsequently aggregating their outputs through a majority voting mechanism^55,89^. This method is particularly advantageous when the number of training samples is small compared to the dataset size. Additionally, RSS enhances classification performance in datasets with a high degree of feature redundancy by reducing the influence of irrelevant features^95,100^. The process involves randomly selecting feature subsets from the training data, training multiple classifiers, and integrating their classification rules. The final prediction is determined through majority voting, as mathematically represented in Eq. 5^101^.

5 $$\:\beta\:\left(x\right)={argmax}_{y\in\:\{-\text{1,1}\}}\sum\limits_{d}\:{\delta}_{\text{sng}}\left({C}^{b}\left(x\right)\right),y$$.

where $\:\delta\:$ is the Kronecker symbol, $\:y\in\:\{-\text{1,1}\}$ is the decision or class label of the classifier, and $\:{C}^{b}\left(x\right)$ is the classification integration (C = 1, 2, …, C).

### Statistical evaluation of the developed models

In this study, the performance of the developed models was evaluated using multiple statistical measures, including coefficient of determination (R²), Nash-Sutcliffe efficiency (NSE), Willmott’s index of agreement (d), mean absolute error (MAE), root mean square error (RMSE), root relative squared error (RRSE) and relative Absolute Error (RAE) (Eqs. 6–12). along with the visual presentation of line diagram, scatter plo, residual error and Taylor diagram. These metrics provide a comprehensive assessment of the model’s predictive accuracy from different perspectives, ensuring a robust evaluation of its effectiveness. The computation of these performance indicators was carried out using the respective mathematical formulations, as detailed below:

**Table:** 

Statistical measures	Formula	Range	Ideal value	Reference	
Coefficient of determination (R²)	$\:{R}^{2}={\left[\frac{\sum\:_{i=1}^{n}\:\left({\text{T}\text{e}\text{m}\text{p}}_{\text{O}}^{i}-\stackrel{\:}{\stackrel{-}{{{\text{T}\text{e}\text{m}\text{p}}_{\text{O}}^{i}}_{\:}^{\:}}}\right)-\left({\text{T}\text{e}\text{m}\text{p}}_{\text{O}}^{i}-\stackrel{\:}{\stackrel{-}{{{\text{T}\text{e}\text{m}\text{p}}_{\text{P}}^{i}}_{\:}^{\:}}}\right)}{\sqrt{\sum\:_{i=1}^{n}\:{\left({\text{T}\text{e}\text{m}\text{p}}_{\text{O}}^{i}-\stackrel{\:}{\stackrel{-}{{{\text{T}\text{e}\text{m}\text{p}}_{\text{O}}^{i}}_{\:}^{\:}}}\right)}^{2}}\:\sqrt{\sum\:_{i=1}^{n}\:{\left({\text{T}\text{e}\text{m}\text{p}}_{\text{P}}^{i}-\stackrel{\:}{\stackrel{-}{{{\text{T}\text{e}\text{m}\text{p}}_{\text{P}}^{i}}_{\:}^{\:}}}\right)}^{2}}}\right]}^{2}$	0 to 1	1	^86,102,103^	(6)
Nash-Sutcliffe efficiency (NSE)	$\:NSE\:=1-\:\frac{\sum\:_{i=1}^{N}{\left[{\text{T}\text{e}\text{m}\text{p}}_{\text{O}}^{i}-{\text{T}\text{e}\text{m}\text{p}}_{\text{P}}^{i}\right]}^{2}}{\sum\:_{i=1}^{N}{\left[{\text{T}\text{e}\text{m}\text{p}}_{\text{O}}^{i}-\:{\text{T}\text{e}\text{m}\text{p}}_{\text{O}}^{i}\right]}^{2}}$	−∞ to 1	1	^103–105^	(7)
Willmott’s index of agreement (d)	$\:d=1-\frac{\sum\:_{i=1}^{n}\:{\left({\text{T}\text{e}\text{m}\text{p}}_{\text{O}}^{i}-{\text{T}\text{e}\text{m}\text{p}}_{\text{P}}^{i}\right)}^{2}}{\sum\:_{i=1}^{n}\:{\left(\left|{\text{T}\text{e}\text{m}\text{p}}_{\text{P}}^{i}-\stackrel{-}{{\text{T}\text{e}\text{m}\text{p}}_{\text{O}}^{i}}\right|+\left|{\text{T}\text{e}\text{m}\text{p}}_{\text{O}}^{i}-\stackrel{-}{{\text{T}\text{e}\text{m}\text{p}}_{\text{O}}^{i}}\right|\right)}^{2}}$	0 to 1	1	^61^	(8)
Mean absolute error (MAE)	$\:MAE=\frac{1}{N}\sum\:_{i=1}^{n}\:\left|{\text{T}\text{e}\text{m}\text{p}}_{\text{P}}^{i}-{\text{T}\text{e}\text{m}\text{p}}_{\text{O}}^{i}\right|$	0 to ∞	0	^73,103^	(9)
Root mean square error (RMSE)	$\:RMSE=\sqrt{\frac{1}{N}\sum\:_{i=1}^{N}{\left({\text{T}\text{e}\text{m}\text{p}}_{\text{O}}^{i}-{\text{T}\text{e}\text{m}\text{p}}_{\text{P}}^{i}\right)}^{2}}$	0 to ∞	0	^73,103,106–108^	(10)
Root relative squared error (RRSE)	$\:RRSE=\frac{\sqrt{\sum\:_{i=1}^{N}\:{\left({\text{T}\text{e}\text{m}\text{p}}_{\text{P}}^{i}-\stackrel{\:}{{\text{T}\text{e}\text{m}\text{p}}_{\text{O}}^{i}}\right)}^{2}}}{\sqrt{\sum\:_{i=1}^{N}\:{\left({\text{T}\text{e}\text{m}\text{p}}_{\text{O}}^{i}-\stackrel{-}{{{\text{T}\text{e}\text{m}\text{p}}_{\text{O}}^{i}}_{\:}^{\:}}\right)}^{2}}}$	0 to ∞	0	^73,103^	(11)
Relative Absolute Error (RAE)	$\:RAE=\frac{\sum\:_{i=1}^{n}\:\left|{\text{T}\text{e}\text{m}\text{p}}_{\text{P}}^{i}-{\text{T}\text{e}\text{m}\text{p}}_{\text{O}}^{i}\right|}{\sum\:_{i=1}^{n}\:\left|\stackrel{\:}{{\text{T}\text{e}\text{m}\text{p}}_{\text{O}}^{i}}-\stackrel{-}{{{\text{T}\text{e}\text{m}\text{p}}_{\text{O}}^{i}}_{\:}^{\:}}\right|}$	0 to ∞	0	^73,103,106,109^	(12)

where, $\:{\text{T}\text{e}\text{m}\text{p}}_{\text{P}}^{i}$ and $\:{\text{T}\text{e}\text{m}\text{p}}_{\text{O}}^{i}$ are the actual and forecasting air temperature for i^th^ observation While $\:\stackrel{\:}{\stackrel{-}{{{\text{T}\text{e}\text{m}\text{p}}_{\text{O}}^{i}}_{\:}^{\:}}}$, and $\:\stackrel{\:}{\stackrel{-}{{{\text{T}\text{e}\text{m}\text{p}}_{\text{P}}^{i}}_{\:}^{\:}}}$ are the mean of actual and predicted value, and N is the total number of observations.

The coefficient of determination (*R*^2^) quantifies the proportion of variance in the dependent variable explained by independent variables, with higher values indicating a better fit. Willmott’s index (*d*) assesses agreement between observed and predicted values, with *d* = 1 indicating perfect alignment. Nash-Sutcliffe Efficiency (*NSE*) of 1 denotes a perfect model, while values near 0 suggest forecastings as accurate as the mean observed data^103,110^. *RMSE* and *MSE* evaluate average squared forecasting errors, whereas *MAE* measures absolute errors^109^. *RRSE* and RAE assess relative forecasting accuracy, with lower values indicating superior performance^103,109^.

## Results

### Selection of inputs

**Fig. 3:**
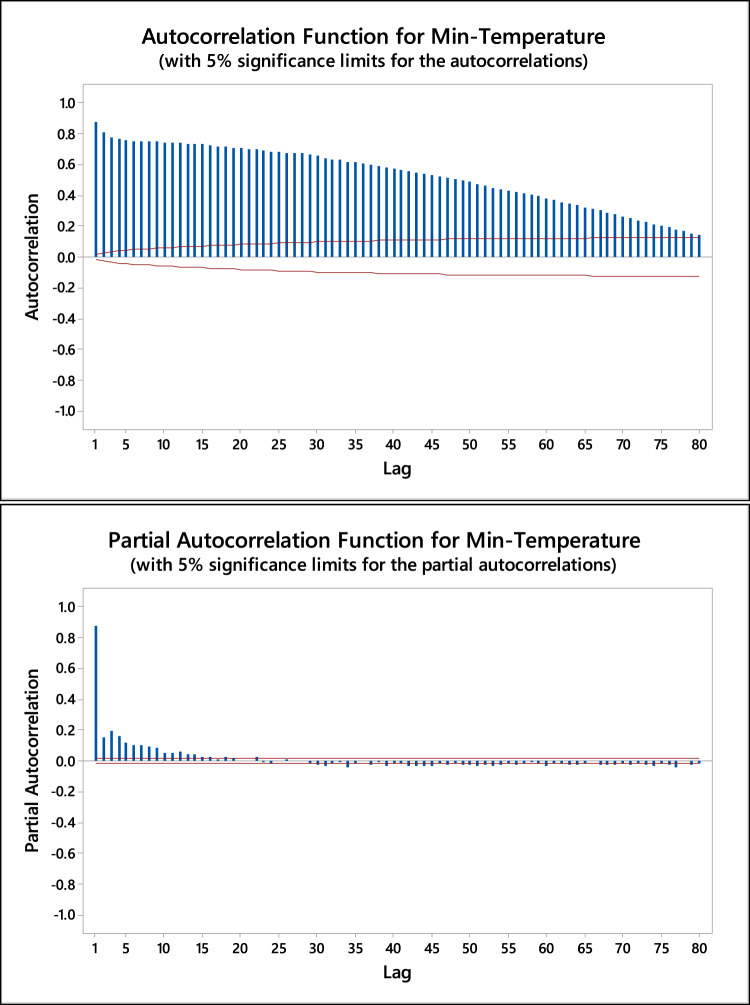
A.C.F. and PACF for minimum temperature using daily and monthly lags.

**Fig. 4:**
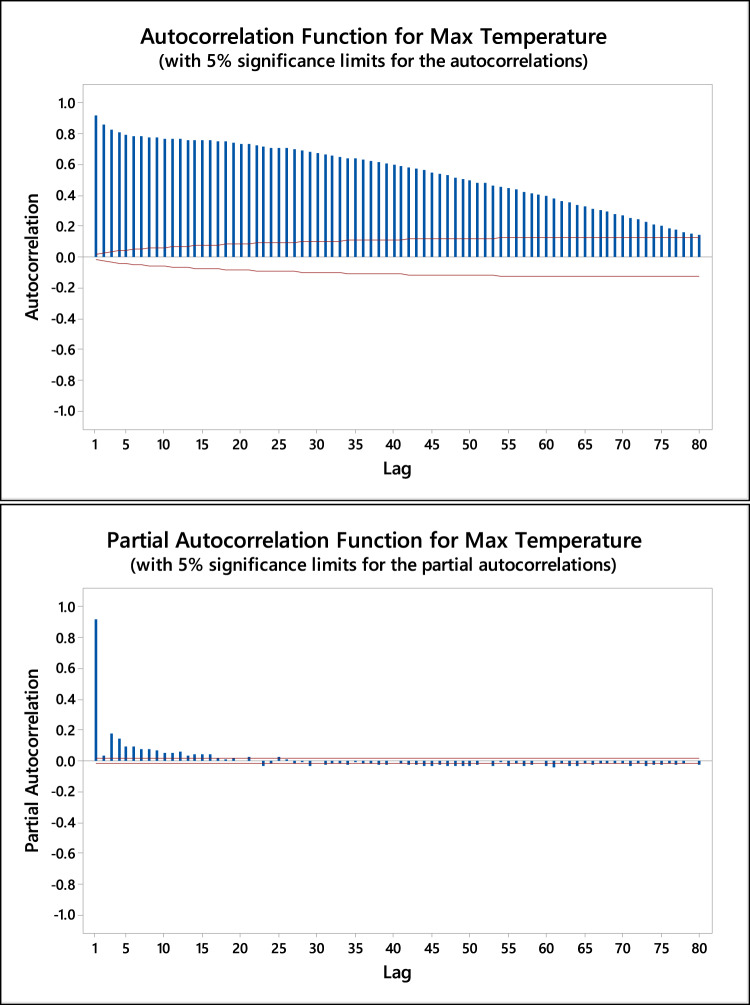
A.C.F. and PACF for maximum temperature using daily and monthly lags.

The time lag for the input variables was determined based on the maximum autocorrelation function (ACF) and partial autocorrelation function (PACF) between the inputs and their respective time lags. Figures 3 and 4 illustrate the ACF and PACF for both minimum and maximum temperatures, with 5% significance limits, respectively. The correlation analysis revealed significant autocorrelations at lag periods ranging from 1 to 8 days for both temperature types. The correlation between T_max_ and its lag values ranged from 0.925 to 0.778, while for T_min_, the correlation ranged from 0.879 to 0.751, both showing a decrease as the lag period increased. Based on these findings, the time lags up to 8 days were selected for further modeling. Additionally, regression analysis was conducted to determine the optimal combination of input variables, incorporating the identified 8-day lag period.

The best subset regression analysis results presented in Table 2 show the performance of various combinations of input variables for predicting minimum air temperature (T_min_). As the number of input variables increases, the mean squared error (MSE) gradually decreases from 7.084 for T_min(t−1)_ alone to 6.214 for the combination of T_min(t−1)_ through T_min(t−8)_. The coefficient of determination (R²) increases slightly, reaching 0.801 for the most comprehensive model (including T_min_ from lags t-1 to t-8), indicating a stronger fit to the observed data. Similarly, the values for Mallows’ Cp, Akaike’s AIC, and Schwarz’s SBC improve, suggesting a better model fit with fewer variables. Despite the improvements, the Amemiya’s PC value remains close to 0.199, showing no significant change across the models. These findings suggest that incorporating additional lags (up to 8 days) yields a marginal but consistent improvement in model performance, without introducing significant complexity or overfitting. The best input combination for forecasting daily minimum temperature were T_min(t−1)_, T_min(t−3)_, T_min(t−4)_, T_min(t−5)_, T_min(t−6)_, T_min(t−7)_, T_min(t−8)_ as shown in Table 2.

Similar to the results for maximum air temperature, the regression analysis for maximum temperature (T_max_) also demonstrates that increasing the number of input variables leads to a slight reduction in mean squared error (MSE), from 8.585 for T_max(t−1)_ to 7.899 for the inclusion of T_max(t−1)_ through T_max(t−8)_ (Table 3). The coefficient of determination (R²) remains consistently high, reaching 0.866, indicating a strong model fit across all combinations. The values of Mallows’ Cp, Akaike’s AIC, and Schwarz’s SBC also improve with additional lags, suggesting better model optimization. However, Amemiya’s PC remains stable at around 0.134, reflecting minimal change with the inclusion of additional lags. These results indicate that extending the time lags up to 8 days enhances model performance without significant overfitting. The best input combination for forecasting daily maximum temperature were T_max (t−1)_, T_max (t−2)_, T_max (t−3)_, T_max (t−4)_, T_max (t−5)_, T_max (t−6)_, T_max (t−8)_ as shown in Table 3.

**Table 2:** Regression analysis using a combination of variables - minimum temperature.

Variables	MSE	*R*²	Mallows’ Cp	Akaike’s AIC	Schwarz’s SBC	Amemiya’s PC
T_min(t−1)_	7.084	0.773	1819.940	25428.592	25443.536	0.227
T_min(t−1)_, T_min(t−8)_	6.501	0.792	602.961	24314.541	24336.956	0.208
T_min(t−1)_, T_min(t−4)_, T_min(t−8)_	6.290	0.799	161.832	23885.822	23915.708	0.201
T_min(t−1)_, T_min(t−4)_, T_min(t−6)_, T_min(t−8)_	6.243	0.800	65.505	23790.280	23827.639	0.200
T_min(t−1)_, T_min(t−3)_, T_min(t−4)_, T_min(t−6)_, T_min(t−8)_	6.230	0.801	39.425	23764.290	23809.120	0.200
Tmin_(t−1)_, T_min(t−3)_, T_min(t−4)_, T_min(t−5)_, T_min(t−7)_, T_min(t−8)_	6.220	0.801	19.252	23744.144	23796.446	0.199
T_min(t−1)_, T_min(t−3)_, T_min(t−4)_, T_min(t−5)_, T_min(t−6)_, T_min(t−7)_, T_min(t−8)_	6.215	0.801	9.280	23734.172	23793.946	0.199
T_min(t−1)_, T_min(t−2)_, T_min(t−3)_, T_min(t−4)_, T_min(t−5)_, T_min(t−6)_, T_min(t−7)_, T_min(t−8)_	6.214	0.801	9.000	23733.891	23801.136	0.199

The best model for the selected selection criterion is displayed in blue.

**Table 3:** Regression analysis using a combination of variables - maximum temperature.

Variables	MSE	*R*²	Mallows’ Cp	Akaike’s AIC	Schwarz’s SBC	Amemiya’s PC
T_max (t−1)_	8.585	0.855	1130.309	27924.126	27939.069	0.145
T_max (t−1)_, T_max (t−8)_	8.130	0.862	383.475	27218.099	27240.514	0.138
T_max (t−1)_, T_max (t−4)_, T_max (t−8)_	8.004	0.865	176.959	27015.874	27045.761	0.135
T_max (t−1)_, T_max (t−2)_, T_max (t−4)_, T_max (t−8)_	7.940	0.866	72.903	26912.756	26950.114	0.134
T_max (t−1)_, T_max (t−2)_, T_max (t−4)_, T_max (t−6)_, T_max (t−8)_	7.909	0.866	23.334	26863.334	26908.164	0.134
T_max (t−1)_, T_max (t−2)_, T_max (t−3)_, T_max (t−4)_, T_max (t−6)_, T_max (t−8)_	7.900	0.866	9.520	26849.524	26901.826	0.134
T_max (t−1)_, T_max (t−2)_, T_max (t−3)_, T_max (t−4)_, T_max (t−5)_, T_max (t−6)_, T_max (t−8)_	7.899	0.866	8.030	26848.033	26907.806	0.134
T_max (t−1)_, T_max (t−2)_, T_max (t−3)_, T_max (t−4)_, T_max (t−5)_, T_max (t−6)_, T_max (t−7)_, T_max (t−8)_	7.899	0.866	9.000	26849.002	26916.247	0.134

The best model for the selected selection criterion is displayed in blue.

### Additional analysis of seasonal component

**Fig. 5:**
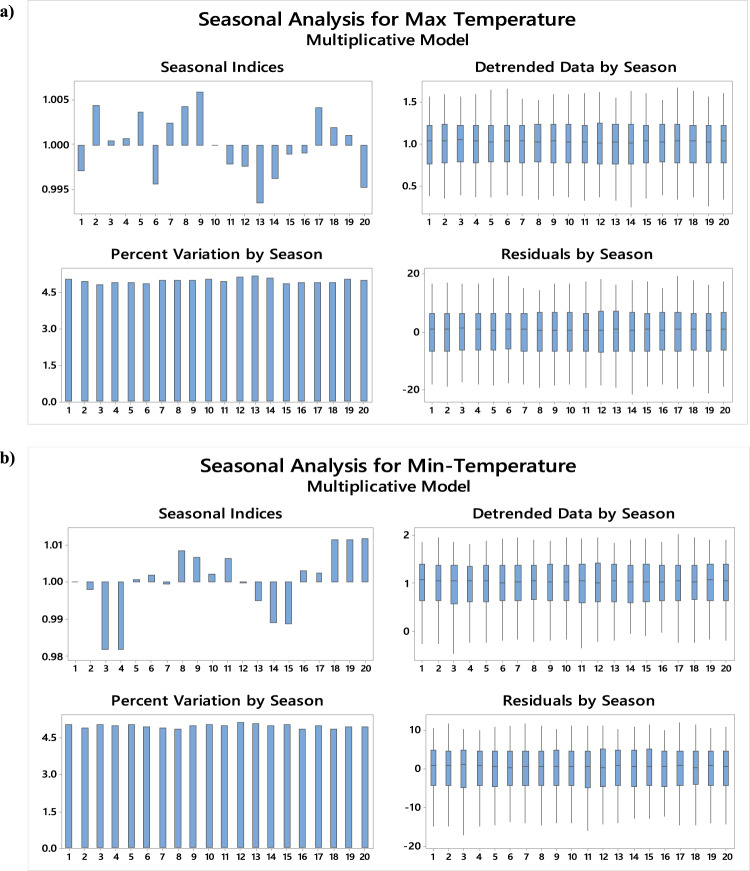
Seasonal (Yearly) analysis for **(a)** maximum and **(b)** minimum temperature.

The seasonal component analysis of the daily maximum and minimum air temperature time series is further explored through four plots in Fig. 5, which include seasonal indices, detrended data by season, percent variation by season, and residuals by season, each showing yearly data. In Fig. 5a, the bar chart (top left) illustrates the variation in seasonal indices over the 20-year period. For maximum temperature, the largest variations are observed in nine years, with the highest bars indicating the greatest disparity for those months. For minimum temperature, the most notable variation occurs in years 18–20. In Fig. 5b, similar trends are observed, with year 13 showing the highest seasonal variation for both maximum and minimum temperatures. The detrended data (top right in Fig. 5a-b), presented as boxplots, indicates minimal change due to the slight trend in the time series, suggesting little effect from detrending. The residuals, shown in the bottom right of Fig. 5(a-b), further confirm consistent seasonal patterns, with yearly fluctuations varying in magnitude, reinforcing the presence of seasonality in the data.

### Minimum air temperature forecasting

Table 4 presents a summary of the prediction performance indices obtained in this study for the predictive models, including Linear Regression, Additive Regression, Random Subspace, M5P, and SVM for minimum temperature forecasting. The graphical representations of the time series are provided in Supplementary Figures S1–S2, while the scatter plots and violin plots with embedded box plots for the training and testing phases are presented in Supplementary Figures S2–S3, respectively. To evaluate the simulation potential of the selected LR, AR, RSS, M5P, and SVM models for minimum air temperature forecasting, the models were trained using 10,219 datasets and tested on 2,768 datasets, with input variables T_min(t−1)_, T_min(t−3)_, T_min(t−4)_, T_min(t−5)_, T_min(t−6)_, T_min(t−7)_, T_min(t−8)_ (Table 2). Additionally, the entire dataset was shuffled and subjected to validation to ensure model robustness. The performance evaluation of various machine learning models for minimum temperature forecasting, as detailed in Table 4, highlights significant variations across training, testing, and validation phases based on multiple statistical indices, including Nash-Sutcliffe Efficiency (NSE), Index of Agreement (d), mean absolute error (MAE), root mean square error (RMSE), relative absolute error (RAE), root relative squared error (RRSE), pearson correlation coefficient (PCC), and coefficient of determination (R²). The NSE values range from a minimum of 0.7506 (Additive Regression, testing) to a maximum of 0.8145 (Random Subspace, training); however, the best-performing model should demonstrate superior performance in both testing and validation phase. In this regard, the M5P model exhibited the highest NSE values of 0.7951 in testing and 0.8048 in validation, outperforming all other models. The RMSE values varied between 2.113 (Additive Regression, testing) and 2.749 (Additive Regression, validation), with M5P achieving the lowest RMSE of 2.445 in testing and 2.488 in validation, reinforcing its strong predictive ability. Similarly, M5P demonstrated a high index of agreement (d) of 0.9411 in testing and 0.9433 in validation, further confirming its reliability in capturing temperature variations. The Pearson Correlation Coefficient (PCC) also indicated M5P as the most effective model, with values of 0.8919 (testing) and 0.8971 (validation), ensuring its ability to capture strong relationships between predicted and actual values. Furthermore, the M5P model recorded one of the lowest error values, with a MAE of 1.899 in testing and 1.952 in validation, while also maintaining the lowest RAE (40.465) and RRSE (45.062) during testing. Although the Random Subspace model showed the highest NSE (0.8145) in training, its performance slightly declined in testing and validation, making M5P the most consistent and reliable model across all phases. In contrast, Additive Regression consistently underperformed across all metrics, with the lowest NSE (0.7506), highest RMSE (2.749), and lowest PCC (0.8671), making it the weakest model. Based on comprehensive performance evaluation across all statistical measures, the models are ranked as follows: M5P > Random Subspace > Linear Regression > SVM > Additive Regression.

**Table 4:** Results of a performance evaluation using the developed ML models during training, testing and validation phase for minimum air temperature forecasting.

Model	Dataset	NSE	d	MAE	RMSE	RAE	RRSE	PCC	*R* ^2^
Linear Regression	*Training*	0.8025	0.9424	1.952	2.503	40.506	44.441	0.8958	0.8025
	*Testing*	0.7938	0.9397	1.902	2.453	40.528	45.208	0.8910	0.7938
	*Validation*	0.8022	0.9424	1.953	2.505	40.528	44.470	0.8957	0.8022
Additive Regression	*Training*	0.7675	0.9293	2.158	2.716	44.774	48.219	0.8763	0.7678
	*Testing*	0.7506	0.9250	2.113	2.113	45.041	49.716	0.8671	0.7519
	*Validation*	0.7617	0.9296	2.174	2.749	45.111	48.813	0.8728	0.7619
Random Subspace	*Training*	0.8145	0.9436	1.916	2.42	39.765	43.067	0.9041	0.8173
	*Testing*	0.7845	0.9350	1.962	2.507	41.809	46.213	0.8864	0.7857
	*Validation*	0.7865	0.9344	2.051	2.602	42.546	46.202	0.8878	0.7882
M5P	*Training*	0.8061	0.9437	1.945	2.480	40.363	44.033	0.8978	0.8061
	*Testing*	0.7951	0.9411	1.899	2.445	40.465	45.062	0.8919	0.7956
	*Validation*	0.8048	0.9433	1.952	2.488	40.489	40.488	0.8971	0.8048
SVM	*Training*	0.8012	0.9427	1.945	2.511	40.356	44.587	0.8957	0.8022
	*Testing*	0.7920	0.9399	1.899	2.463	40.464	45.405	0.8905	0.7930
	*Validation*	0.8009	0.9427	1.947	2.513	40.393	44.613	0.8955	0.8020

The best-performing machine learning model for forecasting daily minimum air temperature is highlighted in bold blue, while the poorest-performing model is indicated in red.

**Fig. 6:**
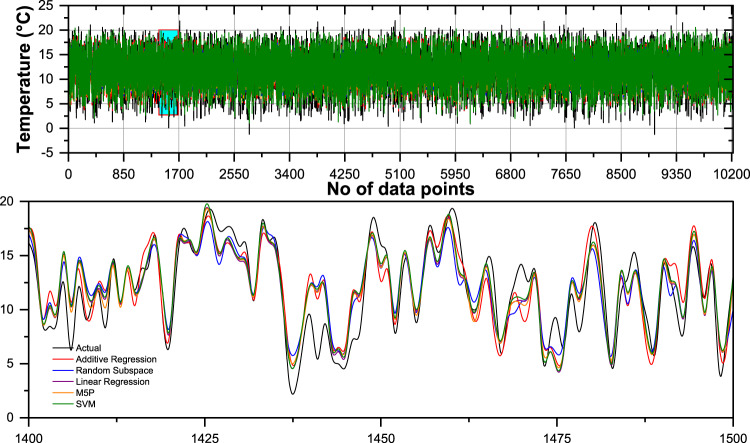
Comparison of daily measured and forecasted minimum temperatures using the most accurate input combination of the developed machine learning algorithms during the validation period. The bottom panel provides a zoomed-in view of the observed and forecasted data.

For enhanced visualization and comparative analysis of the models, a line diagram, scatter plots illustrating the relationship between actual values and forecasted temperature with a 95% prediction band, and a histogram incorporating the Gaussian kernel density function are employed using the most accurate input combinations. Figure 6 present a comparative analysis of the daily measured and forecasted minimum air temperatures utilizing the most accurate input combination of the developed machine learning algorithms during the validation period. Additionally, the bottom panel offers a magnified view, providing a detailed representation of the observed and predicted temperature variations. Overall, while all models generally follow the trend of the actual values, they exhibit deviations in peaks and troughs, with some models such as M5P and Random Subspace appearing to track the actual values more closely in certain segments. Figure 6 indicates their tendency to overestimate or underestimate the actual values. The residuals of Additive Regression show a slight positive bias with a mean bias error of 0.003, suggesting minor overestimation. Random Subspace and Linear Regression have mean bias errors of −0.002 and − 0.001, respectively, indicating a very slight underestimation. The M5P model has a mean bias error of 0, meaning it exhibits no systematic bias in its predictions. Among all models, SVM has the highest mean bias error at 0.169, suggesting a noticeable overestimation compared to the actual values.

**Fig. 7:**
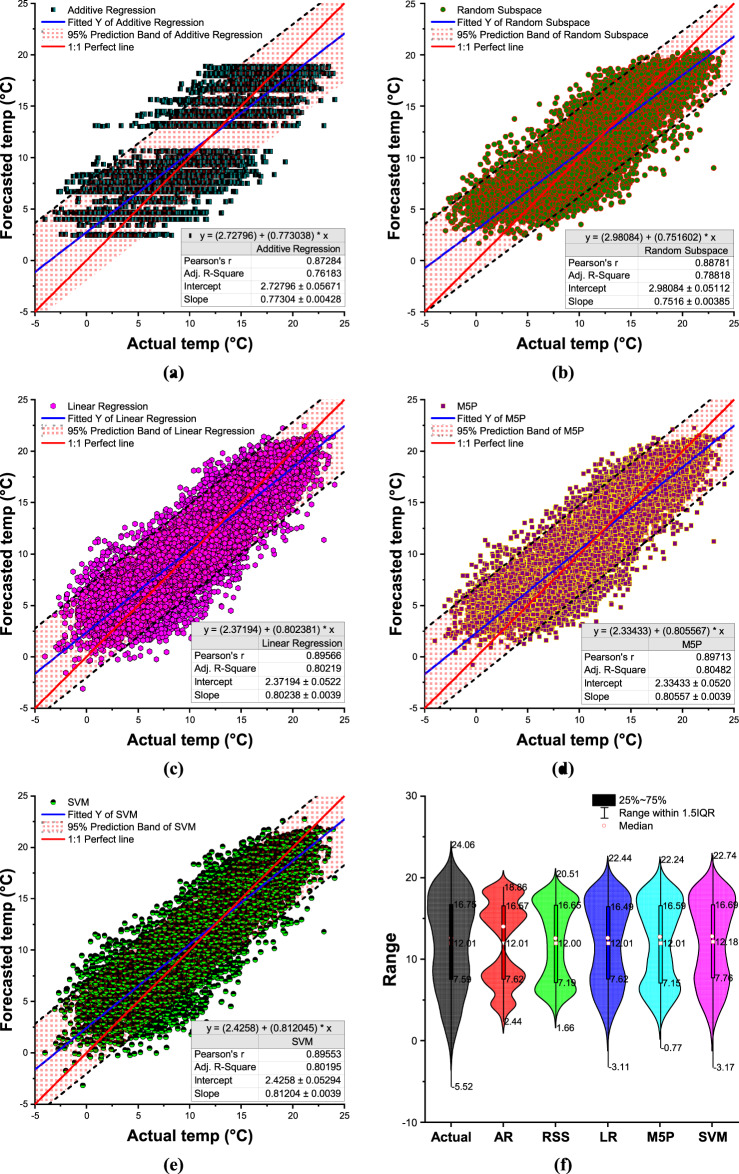
Scatter plots **(a–e)** and a violin plot with a box plot **(f)** comparing actual and forecasted daily minimum temperature values using the most accurate input combination of the developed ML algorithms during the validation period.

While Fig. 7(a-e) shows the scatter plots with 95% prediction band and a violin plot with a box plot Fig. 7(f) comparing actual and forecasted daily minimum temperature values for during the training period and testing phase, respectively. The 95% prediction band is the area in which you expect 95% of all data points to fall. In contrast, the 95% confidence band is the area that has a 95% chance of containing the true regression line. Additive regression (AR) failed to predict the highest and lowest values ​​in both cases (both minimum and maximum temperatures) during the training, testing and validation periods. The violin plot compares the distribution of actual and predicted temperature ranges across different machine learning models, including AR, RSS, LR, M5P, and SVM (Fig. 7(f)). The figure visualizes the spread of temperature variations, median values, and interquartile ranges (IQR), which help assess the accuracy of each model in capturing temperature fluctuations. The actual temperature range spans from 5.52 to 24.06, with a median of 12.01 and an interquartile range between 7.59 and 16.75. Among the predictive models, M5P demonstrates the closest resemblance to the actual distribution. It has a range of 0.77 to 22.24, a median value of 12.01, and an IQR between 7.15 and 16.59. These values indicate that M5P effectively captures the temperature variations while maintaining a distribution similar to the actual data.

Other models, such as AR and RSS, exhibit narrower distributions, with AR ranging from 2.44 to 18.67 and RSS from 1.66 to 20.51. Their inability to capture extreme values suggests that they may struggle to predict temperature variations accurately. Linear Regression LR follows closely behind M5P, with a range of 3.11 to 22.44, but it slightly underestimates temperature extremes. The SVM model, although similar to LR, has a slightly wider range from − 3.17 to 22.74, indicating occasional under-predictions. Overall, M5P emerges as the best-performing model, as it aligns most closely with the actual temperature range while maintaining a balanced distribution and accurate median prediction. The results suggest that M5P provides the most reliable temperature forecasts among the evaluated models.

**Fig. 8:**
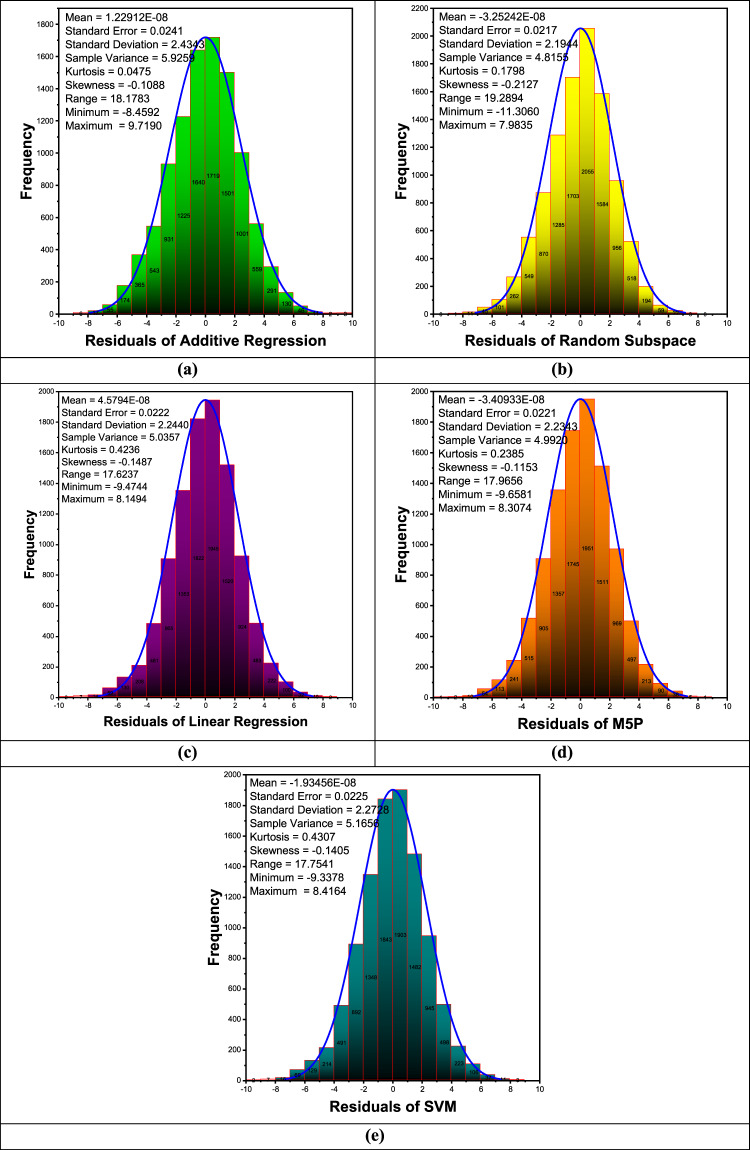
The histogram and Gaussian kernel density function for daily minimum temperature forecasting during the validation phase.

Figure 8(a-e) presents histogram plots illustrating the forecasting error distribution during the validation phase. These plots provide a visual representation of the error distribution by depicting the frequency of error values within predefined intervals. Additionally, a Gaussian kernel density function is incorporated to assess the normality of the error distribution, offering insights into the overall error characteristics and potential deviations from a normal distribution. The residual analysis of predictive models reveals their accuracy and distribution. The mean residual values for all models are close to zero, indicating minimal systematic bias. Standard deviation and variance values show that Additive Regression has the highest variability, while Random Subspace has the lowest, suggesting that Random Subspace provides more consistent predictions. Kurtosis values are positive but relatively low, indicating a distribution close to normal with slightly heavier tails. Skewness values are negative for all models, suggesting a slight leftward asymmetry in residual distributions, though the magnitude remains small. The range of residuals is highest for Random Subspace (19.2894) and lowest for Linear Regression (17.6237), indicating that Random Subspace has the widest spread of errors. Minimum residual values show that Random Subspace underestimates the most (−11.3060), while Additive Regression has the highest overestimation (9.7190). Overall, all models exhibit relatively small residual errors, with some variation in distribution and spread. Additive Regression and Linear Regression display slightly higher residual variability, whereas Random Subspace and M5P show more constrained predictions. SVM has moderate error dispersion but does not exhibit extreme bias. These insights help in selecting a model based on error consistency and bias considerations.

**Fig. 9:**
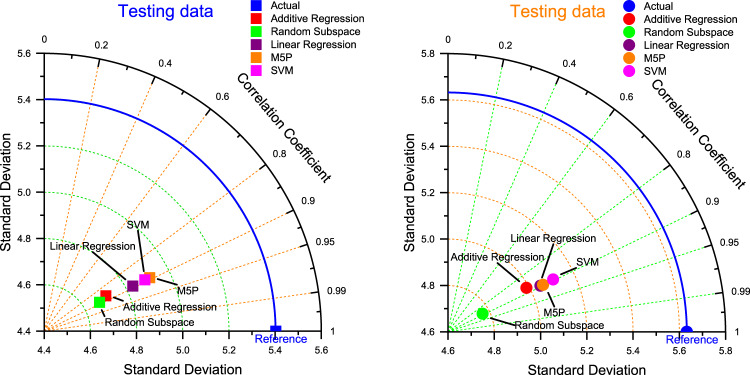
Taylor diagram of predicting daily minimum temperature amounts using the most accurate models in the testing and validation period.

The results of the Table 4; Figs. 6, 7 and 8 along with the Taylor diagram confirm that the M5P has outperformed other benchmark models in forecasting accuracy across all the phase (testing and validation phase) (Fig. 9).

### Maximum air temperature forecasting

The performance evaluation of the developed machine learning (ML) models for maximum air temperature forecasting, as presented in Table 5, indicates that the models exhibit varying degrees of accuracy based on statistical indices across training, testing, and validation phases. Whereas the graphical representation of the time series are given in ‘Supplementary material’ Supplementary Figure S5-S6, scatter plot and violin plot with a box plot are given in ‘Supplementary material’ Supplementary Figure S7-S8 for training and testing phase, respectively. Among all models, M5P demonstrated the best overall performance, with the highest NSE values of 0.8761, 0.8473, and 0.8720 for training, testing, and validation, respectively. It also exhibited the lowest RMSE values (2.7248, 2.9027, and 2.7696) and MAE values (1.9555, 2.0773, and 1.9867) across the three phases. Additionally, M5P achieved the highest PCC values (0.9360, 0.9206, and 0.9338), confirming its superior predictive capability. In contrast, the Additive Regression model exhibited the weakest performance, with the lowest NSE values (0.8362, 0.8072, and 0.8305) and the highest RMSE values (3.1329, 3.2611, and 3.1872). The model also recorded the highest RAE (34.4855, 37.3644, and 35.0419) and RRSE (40.467, 43.512, and 41.165), indicating significant deviations in predicted values compared to observed data. Furthermore, its PCC values (0.9147, 0.8989, and 0.9113) were lower than those of the other models, reinforcing its relatively poorer predictive accuracy.

The performance of Linear Regression, Random Subspace, and Support Vector Machine (SVM) models was intermediate, with NSE values ranging from 0.8466 to 0.8708, RMSE values between 2.7828 and 2.9890, and PCC values varying from 0.9080 to 0.9338. These models demonstrated moderate forecasting accuracy but were outperformed by M5P in all statistical aspects. Overall, M5P emerged as the most effective model for maximum temperature forecasting, achieving the highest accuracy and lowest error rates, while Additive Regression performed the poorest, displaying the highest prediction errors and lowest efficiency. The models ranked based on performance are: M5P (Best) > Linear Regression > SVM > Random Subspace > Additive Regression (Worst), with M5P showing the highest accuracy and Additive Regression the poorest performance.

**Table 5:** Results of a performance evaluation using the developed ML models during training, testing and validation phase for maximum air temperature forecasting.

Model	Date set	NSE	d	MAE	RMSE	RAE	RRSE	PCC	R^2^
Linear Regression	*Training*	0.8708	0.9644	1.9981	2.7828	29.7166	35.945	0.9332	0.8708
	*Testing*	0.8466	0.9574	2.0832	2.9090	32.0717	38.815	0.9202	0.8467
	*Validation*	0.8705	0.9643	2.0001	2.7861	29.7428	35.985	0.9330	0.8705
Additive Regression	*Training*	0.8362	0.9529	2.3187	3.1329	34.4855	40.467	0.9147	0.8367
	*Testing*	0.8072	0.9445	2.4270	3.2611	37.3644	43.512	0.8989	0.8079
	*Validation*	0.8305	0.9520	2.3565	3.1872	35.0419	41.165	0.9113	0.8305
Random Subspace	*Training*	0.8675	0.9618	2.0719	2.8181	30.8150	36.402	0.9323	0.8693
	*Testing*	0.8231	0.9481	2.2817	3.1238	35.1278	41.680	0.9080	0.8244
	*Validation*	0.8209	0.9570	2.1836	2.9890	32.4712	38.606	0.9230	0.8519
M5P	Training	0.8761	0.9660	1.9555	2.7248	29.0838	35.196	0.9360	0.8761
	Testing	0.8473	0.9578	2.0773	2.9027	31.9806	38.731	0.9206	0.8475
	Validation	0.8720	0.9649	1.9867	2.7696	29.5440	35.773	0.9338	0.8720
SVM	*Training*	0.8696	0.9645	1.9861	2.7952	29.5388	36.106	0.9328	0.8701
	*Testing*	0.8453	0.9575	2.0756	2.9215	31.9543	38.982	0.9199	0.8463
	*Validation*	0.8694	0.9644	1.9881	2.7975	29.5648	36.133	0.9327	0.8699

The best-performing machine learning model for forecasting daily minimum air temperature is highlighted in bold blue, while the poorest-performing model is indicated in red.

**Fig. 10:**
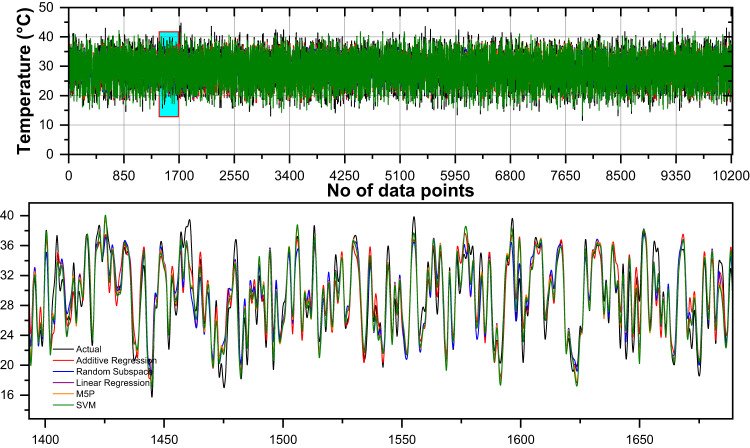
Comparison of daily measured and forecasted maximum temperatures using the most accurate input combination of the developed machine learning algorithms during the validation period. The bottom panel provides a zoomed-in view of the observed and forecasted data.

The comparison of daily measured and forecasted maximum temperatures using different machine learning models during the validation period reveals varying degrees of prediction accuracy (Fig. 10). The mean bias error (MBE) provides insight into the tendency of each model to overestimate or underestimate temperature values. Among the models, linear regression demonstrates the most balanced performance with an MBE of zero, indicating no systematic bias in its predictions. This suggests that, on average, the model neither over-predicts nor under-predicts the actual temperatures, making it highly reliable.

The random subspace and additive regression models exhibit slight underestimation, with MBEs of −0.006 and − 0.022, respectively. Their minor negative bias suggests that while they closely follow the actual temperature trends, they tend to predict slightly lower values than observed. Similarly, the M5P model shows a small positive bias of 0.006, implying a slight overestimation of temperatures. These minimal deviations indicate that all three models perform well, with only marginal tendencies to either underestimate or overestimate.

In contrast, the SVM model shows the most significant bias, with an MBE of −0.153, indicating a considerable underestimation of maximum temperatures. This suggests that the SVM model struggles to fully capture the variability in temperature data, leading to consistent under-prediction. The results highlight that while most models perform well with minimal bias, SVM is less reliable in accurately forecasting maximum temperatures during the validation period.

**Fig. 11:**
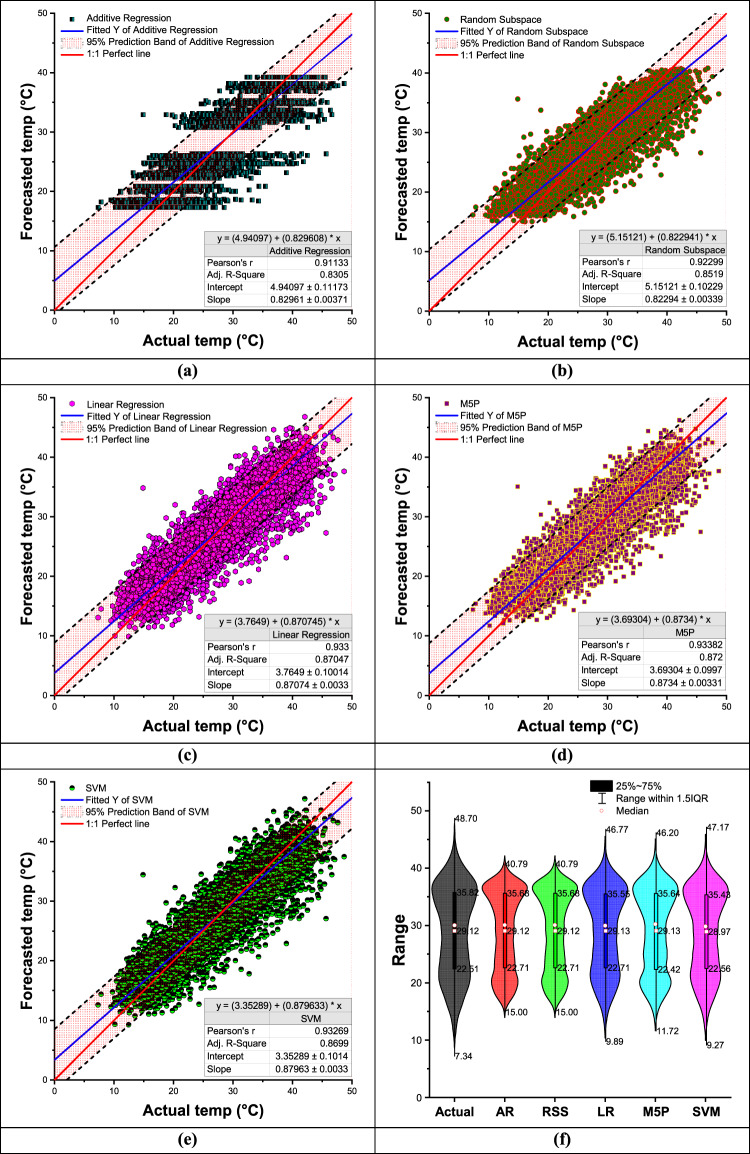
Scatter plots **(a–e)** and a violin plot with a box plot **(f)** comparing actual and forecasted daily maximum temperature values using the most accurate input combination of the developed ML algorithms during the validation period.

Figure 11(a–e) presents scatter plots with a 95% prediction band, while Fig. 11(f) displays a violin plot combined with a box plot, comparing actual and forecasted daily maximum temperature values during the training and testing phases. The 95% prediction band represents the range where 95% of the data points are expected to fall, whereas the 95% confidence band indicates the range with a 95% probability of containing the true regression line. Additive Regression (AR) demonstrated limitations in accurately forecasting both the highest and lowest temperature values during the training, testing, and validation periods, failing to capture the full range of temperature variability while the other models very closed to line 1:1. The violin plot presents a comparative analysis of the actual and predicted temperature ranges across different machine learning models, including Additive Regression (AR), Random Subspace (RSS), Linear Regression (LR), M5P, and Support Vector Machine (SVM) (Fig. 11(f)). The plot illustrates the distribution, median, and interquartile range (IQR) of temperature predictions, providing insights into the models’ accuracy and performance. The actual temperature range varies from 7.34 to 48.70, with a median value of 29.12 and an interquartile range between 22.51 and 35.82. Among the models, M5P exhibits the closest resemblance to the actual distribution, with a range from 11.72 to 46.20, a median value of 29.13, and an interquartile range between 22.42 and 35.64. This alignment suggests that M5P effectively captures the variability and central tendency of the actual temperature distribution. Comparing the other models, AR and RSS display similar distributions with slightly narrower ranges (15.00 to 40.79) and a median of 29.12, but they fail to capture extreme values as effectively as M5P. Linear Regression (LR) follows closely, with a slightly lower minimum of 9.89 and a comparable IQR (22.71 to 35.55). SVM, on the other hand, demonstrates the least accurate performance, with a range of 9.27 to 47.17 and a median of 28.97, showing a tendency for underestimating temperature variations. Overall, the M5P model stands out as the best-performing model due to its strong alignment with actual temperature values. It maintains a well-balanced distribution, accurately capturing both the median and spread of the data while encompassing extreme values more effectively than the other models. This makes M5P the most reliable choice for temperature prediction in this scenario.

**Fig. 12:**
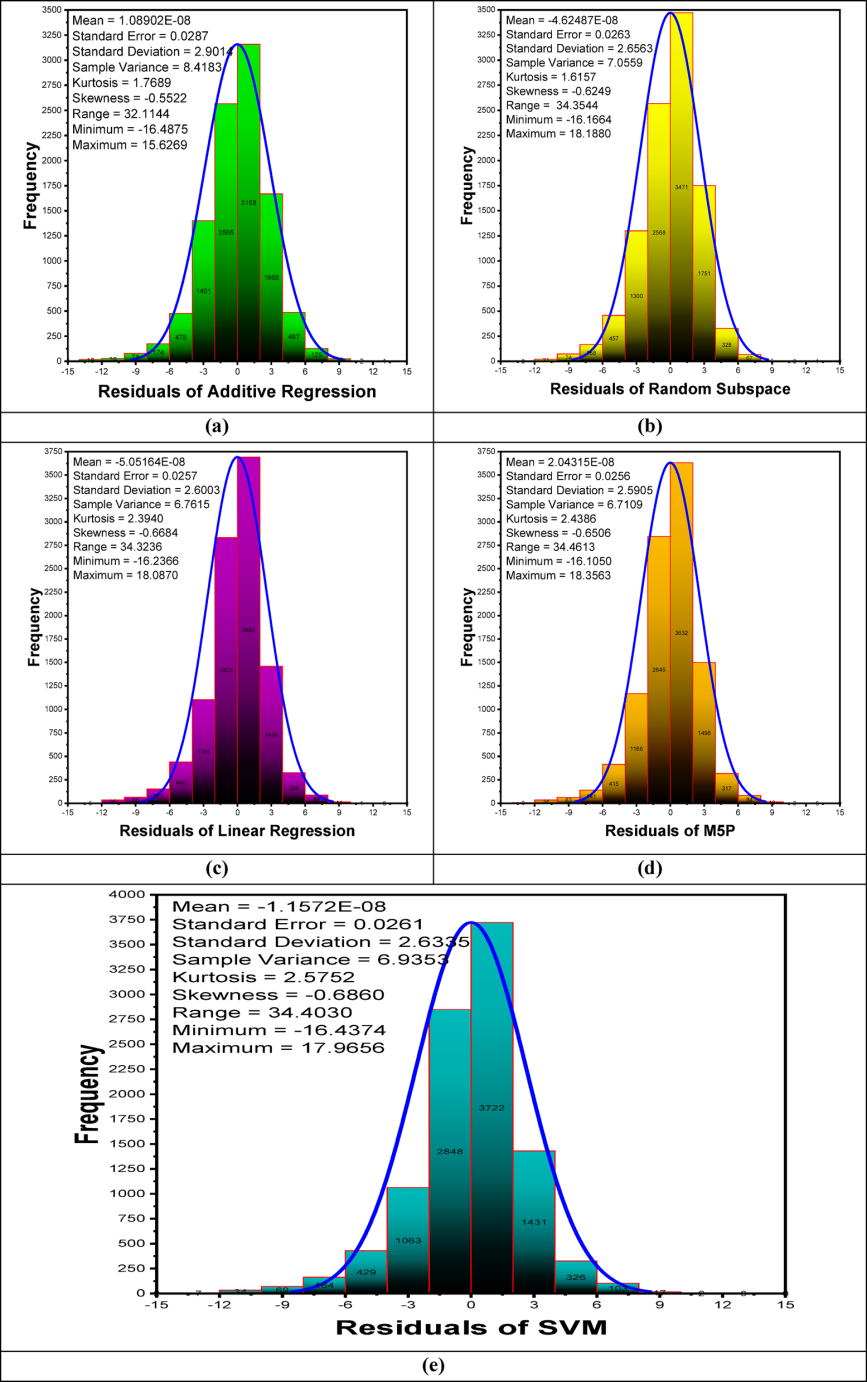
The histogram and Gaussian kernel density function of residual error for daily maximum temperature forecasting during the validation phase.

Figure 12(a-e) presents histogram plots illustrating the forecasting residual error distribution during the validation phase. The residual analysis of predictive models for maximum temperature forecasting reveals key insights into their performance. The mean residual values for all models are close to zero, indicating minimal systematic bias. Standard deviation and variance values suggest that Additive Regression has the highest variability, while M5P has the lowest, implying that M5P provides more consistent predictions. Kurtosis values indicate that all residual distributions exhibit slightly heavier tails than a normal distribution, with SVM showing the highest kurtosis, suggesting more extreme residual values. The negative skewness observed across all models indicates a slight tendency toward underestimation, with SVM having the most pronounced leftward skew. The range of residuals is widest for M5P (34.4613) and narrowest for Additive Regression (32.1144), demonstrating varying degrees of error spread among models. The minimum and maximum residual values suggest that Random Subspace exhibits the most significant overestimation, while Additive Regression shows the least. Overall, M5P demonstrates lower error dispersion and variance, indicating more stable performance, while SVM and Linear Regression display higher deviations. These findings highlight M5P as the most reliable model for maximum temperature forecasting due to its minimal variance and balanced error distribution.

**Fig. 13:**
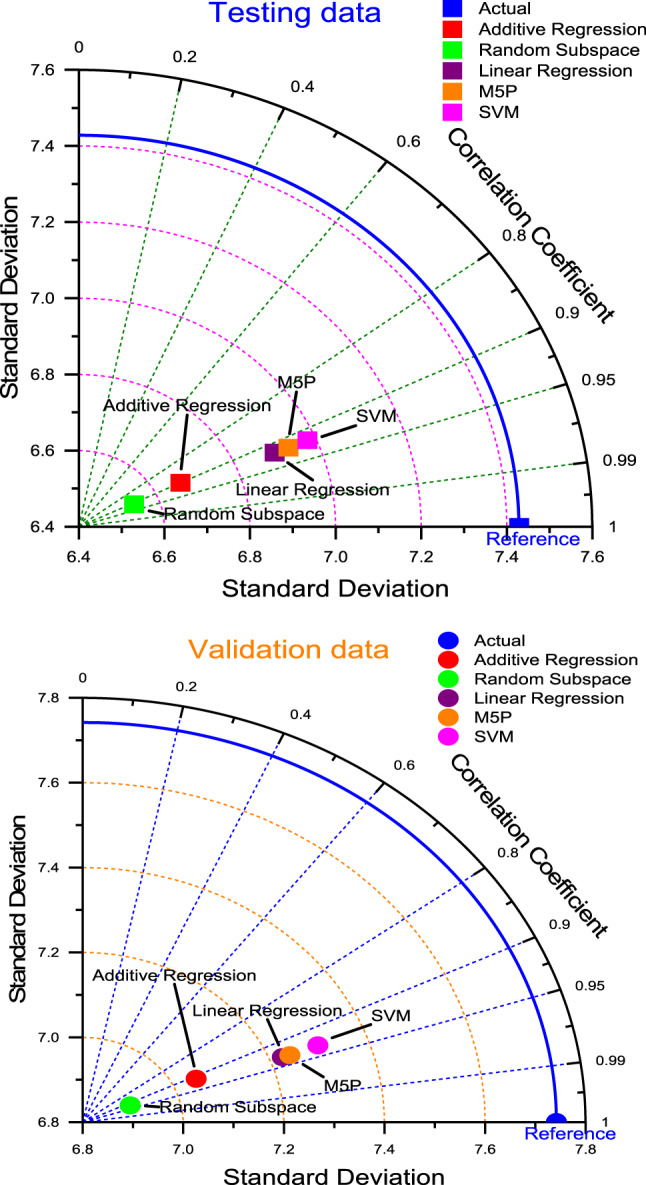
Taylor diagram of predicting daily maximum temperature amounts using the most accurate models in the testing and validation phase.

The results presented in Table 5; Figs. 10, 11 and 12, in conjunction with the Taylor diagram, demonstrate that the M5P model exhibits superior forecasting accuracy compared to other benchmark models across all phases, including both testing and validation (Fig. 13).

### Statistical comparison between the predictive models for minimum air temperature forecasting based Friedman ANOVA

The Friedman test is a non-parametric statistical test used to compare multiple models (or treatments) across multiple datasets (or repeated measures). It helps determine whether at least one model significantly differs in performance from the others. Table 6 shows the descriptive statistics for the Friedman ANOVA analysis on minimum temperature forecasting provide insights into the distribution of predictions across different models. The actual minimum temperatures range from − 5.517 °C to 24.06 °C, with a median of 12.616 °C. Among the models, Additive Regression exhibits the highest median (14.002 °C), indicating a tendency to overestimate compared to actual values, whereas Linear Regression and SVM have medians (12.607 °C and 12.81 °C, respectively) closer to the actual median, suggesting a more balanced prediction. Random Subspace shows a wider range (1.662 °C to 20.505 °C) than most models, indicating higher variability in predictions. The first (Q1) and third (Q3) quartiles across models also highlight variations in predictive dispersion, with M5P and Additive Regression having narrower interquartile ranges compared to others. The maximum and minimum values of models further indicate their ability to capture extreme temperature values, with Linear Regression and SVM capturing a broader range, whereas Additive Regression tends to be more conservative. Overall, the differences in median values and variability suggest notable performance variations, which align with the Friedman test’s objective of assessing statistical differences in model forecasting ability.

**Table 6:** Descriptive statistics of Friedman ANOVA for minimum air temperature forecasting.

	N	Min	Q1	Median	Q3	Max
**Actual**	10,219	−5.517	7.593	12.616	16.747	24.060
**Additive Regression**	10,219	2.435	7.615	14.002	16.570	18.859
**Random Subspace**	10,219	1.662	7.185	12.572	16.654	20.505
**Linear Regression**	10,219	−3.107	7.621	12.607	16.486	22.437
**M5P**	10,219	−0.774	7.145	12.743	16.585	22.240
**SVM**	10,219	−3.166	7.760	12.810	16.691	22.735

N = no of data point, Q1 = first quartile and Q3 = third quartile and Min = minimum value and Max = maximum value of minimum air temperature.

The results of the Friedman test, the significance of the p-value is assessed to determine whether differences exist among the models. If the p-value is less than the significance level (α), typically 0.05, the null hypothesis (H₀), which assumes that all models perform equally, is rejected. This indicates that at least one model exhibits a statistically significant difference in performance compared to the others, suggesting variability in predictive accuracy. Conversely, if the p-value is greater than or equal to α, the null hypothesis is not rejected, implying that there is no statistically significant difference among the models. In this case, the models exhibit similar predictive capabilities based on rankings, and any observed differences in performance are likely due to random variations rather than inherent model superiority.

**Table 7:** ANOVA Post-hoc analysis (Dunn’s Test) results for additive regression, random subspace, linear regression, M5P, and SVM techniques during the validation phase in minimum air temperature.

	Sum Rank Diff	Z	Prob	Sig
**“Actual” “Additive Regression"**	1669	6.24027	6.5524E-9	1
**“Actual” “Random Subspace"**	2704	10.11005	7.47853E-23	1
**“Actual” “Linear Regression"**	6142.5	22.96635	1.51748E-115	1
**“Actual” “M5P"**	3286.5	12.28798	1.57654E-33	1
**“Actual” “SVM"**	−7055	26.37812	3.66447E-152	1
**“Additive Regression” “Random Subspace"**	1035	3.86979	0.00163	1
**“Additive Regression” “Linear Regression"**	4473.5	16.72608	1.26938E-61	1
**“Additive Regression” “M5P"**	1617.5	6.04771	2.20377E-8	1
**“Additive Regression” “SVM"**	−8724	32.61838	3.37606E-232	1
**“Random Subspace” “Linear Regression"**	3438.5	12.85629	1.18931E-36	1
**“Random Subspace” “M5P"**	582.5	2.17792	0.44118	0
**“Random Subspace” “SVM"**	−9759	36.48817	2.56222E-290	1
**“Linear Regression” “M5P"**	−2856	10.67837	1.92761E-25	1
**“Linear Regression” “SVM"**	−13197.5	49.34447	0	1
**“M5P” “SVM"**	−10341.5	38.6661	0	1

Sig equals 1 indicates that the difference of the means is significant at the 0.05 level. Sig equals 0 indicates that the difference of the means is NOT significant at the 0.05 level.

Table 7 shows is the result of Dunn’s Test, a post-hoc analysis typically used after a significant Friedman Test to determine which specific pairs of models exhibit significant differences in their performance. The columns in the table represent the Sum Rank Difference, Z-score, p-value (Prob), and Significance (Sig) between different model pairs. From the results, we observe that most model comparisons show significant differences, as indicated by very low p-values (often much smaller than 0.05). For instance, the comparison between “Actual” and “Linear Regression” has a p-value of 1.51E-115, which is an extremely small value, strongly rejecting the null hypothesis of no difference. Similarly, the comparison between “Actual” and “SVM” has a very large negative Sum Rank Difference (−7055), with a high Z-score (26.37812) and an extremely small p-value (3.66E-152), further confirming substantial differences in their performance.

In contrast, some comparisons do not show significant differences. For instance, the “Random Subspace” vs. “M5P” comparison has a p-value of 0.44118, which is greater than 0.05, meaning that their performance is statistically similar. The same applies to the “M5P” vs. “SVM” comparison, which has a p-value of 0.0, suggesting that these models are significantly different from each other. A notable trend in the results is that SVM appears to be significantly different from most models, often with large negative Sum Rank Differences, indicating poorer performance relative to others. Conversely, Linear Regression and Additive Regression seem to perform distinctly better than SVM and some other models. Overall, the Dunn’s Test results confirm that the models do not perform equally, with several significant differences among them. The results suggest that some models (e.g., Linear Regression, Additive Regression) perform considerably differently from others (e.g., SVM). The findings from this test can help in selecting the best-performing model for minimum temperature forecasting.

### Statistical comparison between the predictive models for maximum air temperature forecasting based Friedman ANOVA

Table 8 shows the descriptive statistics for maximum temperature forecasting using Friedman ANOVA reveal significant variations in model predictions compared to actual values. The actual temperature ranges from 7.34 °C to 48.696 °C, with a median of 30.033 °C, serving as the benchmark for evaluating model accuracy. Among the models, Additive Regression exhibits the highest median (32.12 °C) and a relatively high first quartile (23.148 °C), indicating a tendency to slightly overestimate maximum temperatures. Random Subspace (30.077 °C), Linear Regression (29.988 °C), and SVM (29.843 °C) have medians closely aligning with actual values, suggesting better generalization. M5P (30.237 °C) also provides a reasonable approximation, though its wider range (11.722 °C to 46.2 °C) indicates some variability in predictions. The first (Q1) and third (Q3) quartiles across models highlight different levels of dispersion, with Additive Regression having a relatively higher interquartile range. While Random Subspace and Linear Regression exhibit narrower ranges, they still maintain strong alignment with actual temperatures. The broader spread observed in models like M5P and SVM suggests they capture temperature extremes more effectively. These variations in statistical measures reinforce the need for Friedman ANOVA, ensuring that the performance differences among models are statistically validated rather than due to random fluctuations.

**Table 8:** Descriptive statistics of Friedman ANOVA for maximum air temperature forecasting.

	N	Min	Q1	Median	Q3	Max
**Actual**	10,219	7.34	22.508	30.033	35.818	48.696
**Additive Regression**	10,219	17.303	23.148	32.12	34.989	39.221
**Random Subspace**	10,219	15	22.711	30.077	35.676	40.79
**Linear Regression**	10,219	9.889	22.712	29.988	35.553	46.766
**M5P**	10,219	11.722	22.419	30.237	35.644	46.2
**SVM**	10,219	9.268	22.563	29.843	35.432	47.17

N = no of data point, Q1 = first quartile and Q3 = third quartile and Min = minimum value and Max = maximum value of maximum air temperature.

**Table 9:** ANOVA Post-hoc analysis (Dunn’s Test) results for additive regression, random subspace, linear regression, M5P, and SVM techniques during the validation phase in maximum air temperature.

	Sum Rank Diff	Z	Prob	Sig
**“Actual” “Additive Regression"**	−703.5	2.63033	0.12795	0
**“Actual” “Random Subspace"**	−2931	10.95879	9.04504E-27	1
**“Actual” “Linear Regression"**	−3835	14.33878	1.87861E-45	1
**“Actual” “M5P"**	−2963.5	11.0803	2.34509E-27	1
**“Actual” “SVM"**	4985	18.63854	2.34669E-76	1
**“Additive Regression” “Random Subspace"**	−2227.5	8.32846	1.22855E-15	1
**“Additive Regression” “Linear Regression"**	−3131.5	11.70844	1.73066E-30	1
**“Additive Regression” “M5P"**	−2260	8.44997	4.37063E-16	1
**“Additive Regression” “SVM"**	5688.5	21.26888	3.3075E-99	1
**“Random Subspace” “Linear Regression"**	−904	3.37999	0.01087	1
**“Random Subspace” “M5P"**	−32.5	0.12152	1	0
**“Random Subspace” “SVM"**	7916	29.59733	2.42578E-191	1
**“Linear Regression” “M5P"**	871.5	3.25847	0.0168	1
**“Linear Regression” “SVM"**	8820	32.97732	2.5763E-237	1
**“M5P” “SVM"**	7948.5	29.71885	6.57538E-193	1

Sig equals 1 indicates that the difference of the means is significant at the 0.05 level. Sig equals 0 indicates that the difference of the means is NOT significant at the 0.05 level.

The results of the ANOVA post-hoc analysis using Dunn’s Test for the Additive Regression, Random Subspace, Linear Regression, M5P, and SVM techniques provide insights into the statistical significance of pairwise comparisons among these models. Table 9 presents the sum rank differences, Z-values, probabilities (p-values), and significance indicators. From the results, it is evident that “Actual” values compared to Additive Regression show a non-significant difference (*p* = 0.12795), suggesting that Additive Regression does not significantly deviate from the actual values. However, the comparisons between “Actual” and other models, such as Random Subspace (*p* < 0.001), Linear Regression (*p* < 0.001), M5P (*p* < 0.001), and SVM (*p* < 0.001), exhibit highly significant differences, indicating that these models differ considerably from the actual values.

When comparing the Additive Regression model with other models, significant differences are observed against Random Subspace (*p* < 0.001), Linear Regression (*p* < 0.001), M5P (*p* < 0.001), and SVM (*p* < 0.001), implying that Additive Regression produces notably different results from these models. Similarly, Random Subspace and Linear Regression show a significant difference (*p* = 0.01087), highlighting that their predictive behaviors differ.

Interestingly, the comparison between Random Subspace and M5P (*p* = 0.12152) is not statistically significant, indicating that these two models perform similarly. However, all other comparisons, especially those involving SVM, show extremely low p-values (*p* < 0.001), revealing that SVM’s results are significantly different from those of all other models. Notably, the comparisons between SVM and Random Subspace, Linear Regression, and M5P exhibit the highest significance, with p-values reaching as low as 6.57538E-193, emphasizing the stark contrast between SVM and the other techniques.

Overall, the findings suggest that SVM significantly differs from all other models, while Additive Regression is the closest to actual values. Furthermore, Random Subspace and M5P display similarities, whereas Linear Regression shows notable deviations compared to most techniques. These results underline the importance of choosing the right modeling approach depending on the specific application and desired accuracy.

## Discussion

Over the past several years, the availability of air temperature measurements has facilitated extensive research focused on modeling air temperature using machine learning frameworks. The present study aimed to predict maximum (T_max_) and minimum (T_min_) air temperatures using only previously recorded values (lags) of the same variables. A total of five machine learning models—Linear Regression (LR), Additive Regression (AR), Support Vector Machine (SVM), Random Subspace (RSS), and M5P—were applied and compared based on various numerical performance metrics. The results indicated that for T_min_ forecasting, the M5P model demonstrated the best performance (NSE = 0.7951, 0.8048; MAE = 1.899, 2.445; RMSE = 1.952, 2.488 and R^2^ = 0.7956, 0.8048 during testing and validation period, respectively). Similarly, for T_max_ prediction, M5P also outperformed the other models (NSE = 0.8473, 0.8720; MAE = 2.0773, 1.9867; RMSE = 2.9027, 2.7696 and R^2^ = 0.8475, 0.8720 during testing and validation period, respectively). It is crucial to assess the accuracy of air temperature forecasting in the context of previous studies and compare the findings of this study with those reported in the literature. Although limited studies have specifically employed machine learning for air temperature forecasting, numerous studies have demonstrated the successful application of machine learning techniques when incorporating a combination of various meteorological variables. The superiority of M5P model aligned with Achite et al.^111^Mohaghegh et al.^112^ and Anaraki et al.^113^.

Toharudin et al.^114^ compared between the long short-term memory (LSTM) and the Facebook Prophet models for forecasting T_max_ and T_min_. They reported that RMSE values of approximately 1.23 and 0.97 were obtained using the LSTM model, which are less than the values obtained in our study. Chevalier et al.^84^ compared between SVR and MLPNN models for forecasting air temperature up to 12 h in advance and they reported that the SVR was more accurate compared to the MLPNN exhibiting an R and MAE value of approximately 0.955 and 1.906, respectively. The regression equations developed by Gouvas et al.^115^ for predicting T_max_ and T_min_ were found to be robust tools and excellent forecasting accuracies were obtained for each month of the year. For T_max_ forecasting, the R values were ranged from 0.888 to 0.984, while for the T_min_ forecasting, the R values were ranged from 0.921 to 0.984, respectively. These results clearly demonstrated the superiority of the developed regression equations in comparison to the ML models developed in ou present study. In another study, Sekertekin et al.^116^ compared between various ML models manly, the LSTM, Adaptive Neuro-Fuzzy Inference System (ANFIS) with Fuzzy C-Means (ANFIS-FCM), ANFIS with Subtractive Clustering (ANFIS-SC) and ANFIS with Grid Partition (ANFIS-GP) for forecasting daily anf hourly air temperature. Excellent results were obtained using all models and the LSTM was found to be the more accurate exhibiting the best performances with R, RMSE and MAE of approximately 0.970, 1.359, and 0.993, respectively, which were significantly superior to the values obtained in our study. Rezaeian-Zadeh et al.^117^ examined the effectiveness of the multilayer perceptron neural network (MLPNN) and the radial basis function neural network (RBFNN) for forecasting daily maximum (T_max_) and minimum (T_min_) air temperatures. Their study reported excellent predictive performance, with the MLPNN achieving a correlation coefficient (R) of approximately 0.984 and a root mean square error (RMSE) of 1.8, while the RBFNN attained an R value of 0.964 and an RMSE of 2.9. These findings highlight the superior performance of MLPNN and RBFNN in comparison to the models developed in the present study. Chithra et al.^118^ investigated the feasibility of the Multi-Layer Perceptron Neural Network (MLPNN) model for predicting monthly maximum (T_max_) and minimum (T_min_) temperatures. Although the authors did not provide explicit details regarding the model’s fitting capability, the reported root mean square error (RMSE) values were relatively low, indicating satisfactory predictive performance. Specifically, the RMSE values ranged from 0.38 to 0.84 for T_max_ and from 0.47 to 0.93 for T_min_, suggesting that the MLPNN model effectively captured temperature variations with minimal error.

In a recently published, Oloyede et al.^119^ applied five ML models namely, decision tree regression (DT), XGBoost regression, the MLPNN with limited-memory Broyden-Fletcher-Goldfarb-Shanno optimizer, with stochastic gradient optimizer and with Adam optimizer, i.e., MLPNN-LBFGS, MLPNN-SGD, and MLPNN-AOP for predicting daily T_max_ and T_min_. It was found that the MLPNN-LBFGS was the most accurate model and it surpassed all other ML models by significantly reducing the RMSE and MAE values: MAE from 2.184 to 1.412 °C and RMSE from 2.579 to 1.778 °C for T_max_, and the MAE from 0.876 to 0.788 °C (10.05%) and RMSE from 1.225 to 1.127 °C (8.00%) for T_min_, respectively. While the biggest R-value was found to be approximately 0.845. Ramesh and Anitha^120^ applied the multivariate adaptive regression spline (MARS) and the SVR models for forecasting daily T_max_ and T_min_. Obtained results revealed that performances of the MARS were more accurate compared to the SVR with RMSE and MAE of approximately 0.82 and 0.60 for T_min_, 1.19 and 0.87 for T_max_. It is clear for the obtained results that, the proposed MARS was more accurate compared ot our ML models. Mollick et al.^121^ compared between linear regression (LR), Ridge, SVR, RF, and light gradient boosting machine (LGBM), and the Stacking models for forecasting daily T_max_ and T_min_. According to the obtained results, the Stacking model was found to be the more accurate exhibiting R and MAE values of approximately, 0.856 and 1.024, 0.982 and 0.537, for daily T_max_ and T_min_, respectively. by comparison, the proposed Stacking model was more accurate compared to our model for T_max_ and less accurate compared to T_min_.

Finally, the Friedman ANOVA test was employed to see whether the distributions of the estimated and measured data were identical^122,123^. The Friedman ANOVA and Dunn’s post-hoc test results provide substantial evidence of statistically significant differences among the predictive models for temperature forecasting. The analysis reveals that no single model performs uniformly across both minimum and maximum temperature predictions, highlighting the complexities of temperature modeling.

For minimum temperature forecasting, the results indicate that Additive Regression tends to overestimate values, whereas models like Linear Regression and SVM exhibit medians closer to actual values, suggesting relatively better alignment. However, the variability in prediction dispersion, particularly in Random Subspace and M5P, underscores the inconsistencies in their predictive capabilities. The post-hoc analysis further confirms that SVM significantly deviates from all other models, often exhibiting the largest rank differences, suggesting that its performance is considerably distinct, and potentially less reliable, in this context.

Similarly, in maximum temperature forecasting, Additive Regression again exhibits a tendency to overestimate, while models such as Random Subspace, Linear Regression, and SVM demonstrate closer alignment with actual temperature values. However, the statistical significance of the post-hoc comparisons shows that despite their proximity to actual values, many models exhibit significant differences from one another. Notably, Random Subspace and M5P display similar performance, as indicated by their non-significant pairwise difference (*p* > 0.05), while SVM continues to stand out as significantly different from all other models.

From a critical perspective, while statistical significance suggests meaningful differences among models, it does not necessarily imply practical superiority. The effectiveness of a model should be evaluated not only based on its statistical deviations but also on its ability to generalize across different temperature conditions. The stark differences observed in SVM’s results across both minimum and maximum temperature forecasting suggest it may not be a reliable choice for this application. In contrast, Additive Regression, despite its overestimation tendencies, appears to be more stable. The observed similarity between Random Subspace and M5P indicates that these models could be interchangeable in some contexts.

Ultimately, these findings reinforce the necessity of model selection based on the specific requirements of temperature forecasting rather than relying solely on statistical differences. Future research should consider integrating hybrid approaches or ensemble techniques to mitigate individual model weaknesses and enhance overall predictive performance.

A key conclusion from the previously discussed study on T_max_ and T_min_ forecasting using machine learning (ML) is that the developed ML models demonstrated strong predictive capabilities and robustness. In most case studies, these models outperformed those proposed in the present study. However, while our models relied solely on temperature measurements at multiple lag times, a notable limitation of some published studies is their dependence on a combination of multiple meteorological variables to enhance forecasting accuracy. Several researchers have highlighted that solar radiation can be as influential as other meteorological parameters, such as wind speed, humidity, and precipitation, in air temperature prediction.

Despite the challenge of simultaneously collecting diverse weather variables, the modeling framework presented in this study is technically sound, promising, and adaptable to other locations with available T_max_ and T_min_ measurements.

## Conclusion

This study evaluated the predictive capabilities of multiple forecasting models, including Linear Regression (LR), the Autoregressive (AR) model, Random Search (RS), M5P, and Support Vector Machine (SVM), for estimating daily maximum and minimum temperatures. Statistical techniques such as the autocorrelation function (ACF), partial autocorrelation function (PACF), and regression analysis were employed to identify optimal input variable combinations. The results indicate that the M5P model outperforms other models in forecasting both temperature parameters, while LR and SVM also demonstrate competitive accuracy, suggesting their suitability for temperature prediction. Furthermore, incorporating multiple past observations enhances predictive accuracy, underscoring the importance of optimal time lag selection.

The Friedman ANOVA and Dunn’s test confirm statistically significant differences among the models, with Additive Regression exhibiting a tendency to overestimate, whereas LR and SVM align more closely with actual values. Random Subspace and M5P demonstrate higher variability, with SVM differing significantly from other models. For maximum temperature prediction, Random Subspace and M5P show similar performance, while SVM remains an outlier. However, statistical significance does not inherently imply practical superiority, emphasizing the necessity of balancing predictive accuracy and model stability.

To enhance forecasting performance, future research must explore advanced machine learning models, including ensemble techniques such as Random Forest (RF) and XGBoost, in combination with meta-heuristic optimization approaches like Ant Colony Optimization, the Firefly Algorithm, and the Gray Wolf Optimizer. These methods can improve computational efficiency and generalizability. The study’s findings hold significant practical implications for water resource management and agricultural planning, where improved temperature forecasting can facilitate more effective irrigation scheduling, energy consumption planning, and climate adaptation strategies. By leveraging data-driven methodologies, stakeholders can develop more sustainable and efficient approaches to managing environmental and agricultural resources. This research contributes to advancements in predictive modeling, providing valuable insights for future studies in climate forecasting and resource optimization.

## Electronic supplementary material

Below is the link to the electronic supplementary material.

Supplementary Material 1

## Data Availability

The datasets used and/or analysed during the current study available from the corresponding author on reasonable request.
