# Supplementary material for: Air temperature estimation and modeling using data driven techniques based on best subset regression model in Egypt
Source: Sci Rep. 2025 Jun 20;15:20200. doi: 10.1038/s41598-025-06277-2 (PMC12181251; doi:10.1038/s41598-025-06277-2)
Supplement: Supplementary file 1 — Supplementary Material 1 [file 41598_2025_6277_MOESM1_ESM.docx]

**Supplementary Material**

**Supplementary Figure S1.** Comparison of daily measured and forecasted minimum air temperature using the most accurate input combination of the developed machine learning algorithms during the training period. The bottom panel provides a zoomed-in view of the observed and forecasted data.

**Supplementary Figure S2.** Comparison of daily measured and forecasted minimum air temperature using the most accurate input combination of the developed machine learning algorithms during the testing period. The bottom panel provides a zoomed-in view of the observed and forecasted data.

|  |  |
| --- | --- |
| **(a)** | **(b)** |
|  |  |
| **(c)** | **(d)** |
|  |  |
| **(e)** | **(f)** |

**Supplementary Figure S3.** Scatter plots (a–e) and a violin plot with a box plot (f) comparing actual and forecasted daily minimum air temperature values using the most accurate input combination of the developed ML algorithms during the training period.

|  |  |
| --- | --- |
| **(a)** | **(b)** |
|  |  |
| **(c)** | **(d)** |
|  |  |
| **(e)** | **(f)** |

**Supplementary Figure S4.** Scatter plots (a–e) and a violin plot with a box plot (f) comparing actual and forecasted daily minimum air temperature values using the most accurate input combination of the developed ML algorithms during the testing period.

**Supplementary Figure S5.** Comparison of daily measured and forecasted maximum air temperature using the most accurate input combination of the developed machine learning algorithms during the training period. The bottom panel provides a zoomed-in view of the observed and forecasted data.

**Supplementary Figure S6.** Comparison of daily measured and forecasted maximum air temperature using the most accurate input combination of the developed machine learning algorithms during the testing period. The bottom panel provides a zoomed-in view of the observed and forecasted data.

| **** | **** |
| --- | --- |
| **(a)** | **(b)** |
| **** | **** |
| **(c)** | **(d)** |
| **** | **** |
| **(e)** | **(f)** |

**Supplementary Figure S7.** Scatter plots (a–e) and a violin plot with a box plot (f) comparing actual and forecasted daily maximum air temperature values using the most accurate input combination of the developed ML algorithms during the training period.

|  |  |
| --- | --- |
| **(a)** | **(b)** |
|  |  |
| **(c)** | **(d)** |
|  |  |
| **(e)** | **(f)** |

**Supplementary Figure S8.** Scatter plots (a–e) and a violin plot with a box plot (f) comparing actual and forecasted daily maximum air temperature values using the most accurate input combination of the developed ML algorithms during the testing period.
